# Pan‐cancer analyses of bromodomain containing 9 as a novel therapeutic target reveals its diagnostic, prognostic potential and biological mechanism in human tumours

**DOI:** 10.1002/ctm2.1543

**Published:** 2024-02-01

**Authors:** Yu Chen, Zitong Gao, Isam Mohd‐Ibrahim, Hua Yang, Lang Wu, Yuanyuan Fu, Youping Deng

**Affiliations:** ^1^ Department of Quantitative Health Sciences John A. Burns School of Medicine University of Hawaii at Manoa Honolulu Hawaii USA; ^2^ Department of Molecular Biosciences and Bioengineering College of Tropical Agriculture and Human Resources Agricultural Sciences University of Hawaii at Manoa Honolulu Hawaii USA; ^3^ Cancer Epidemiology Division Population Sciences in the Pacific Program University of Hawaii Cancer Center University of Hawaii at Manoa Honolulu Hawaii USA

**Keywords:** BRD9, diagnosis, immune infiltration, m^6^A, PD‐1, prognosis, SMARCD1

## Abstract

**Background:**

Mutations in one or more genes responsible for encoding subunits within the SWItch/Sucrose Non‐Fermentable (SWI/SNF) chromatin‐remodelling complexes are found in approximately 25% of cancer patients. Bromodomain containing 9 (*BRD9*) is a more recently identified protein coding gene, which can encode SWI/SNF chromatin‐remodelling complexes subunits. Although initial evaluations of the potential of BRD9‐based targeted therapy have been explored in the clinical application of a small number of cancer types, more detailed study of the diagnostic and prognostic potential, as well as the detailed biological mechanism of *BRD9* remains unreported.

**Methods:**

We used various bioinformatics tools to generate a comprehensive, pan‐cancer analyses of *BRD9* expression in multiple disease types described in The Cancer Genome Atlas (TCGA). Experimental validation was conducted in tissue microarrays and cell lines derived from lung and colon cancers.

**Results:**

Our study revealed that *BRD9* exhibited elevated expression in a wide range of tumours. Analysis of survival data and DNA methylation for *BRD9* indicated distinct conclusions for multiple tumours. mRNA splicing and molecular binding were involved in the functional mechanism of *BRD9*. *BRD9* may affect cancer progression through different phosphorylation sites or N^6^‐methyladenosine site modifications. *BRD9* could potentially serve as a novel biomarker for diagnosing different cancer types, especially could accurately forecast the prognosis of melanoma patients receiving anti‐programmed cell death 1 immunotherapy. *BRD9* has the potential to serve as a therapeutic target, when pairing with etoposide in patients with melanoma. The BRD9/SMARCD1 axis exhibited promising discriminative performance in forecasting the prognosis of patients afflicted with liver hepatocellular carcinoma (LIHC) and mesothelioma. Additionally, this axis appears to potentially influence the immune response in LIHC by regulating the programmed death‐ligand 1 immune checkpoint. For experimental validation, high expression levels of BRD9 were observed in tumour tissue samples from both lung and colon cancer patients. Knocking down *BRD9* led to the inhibition of lung and colon cancer development, likely via the Wnt/β‐catenin signalling pathway.

**Conclusions:**

These pan‐cancer study revealed the diagnostic and prognostic potential, along with the biological mechanism of BRD9 as a novel therapeutic target in human tumours.

## Graphical abstract

1. The pan‐cancer analyses of bromodomain containing 9 (BRD9) provided a comprehensive perspective for the development of BRD9‐based targeted therapies for a variety of different cancers.
2. BRD9 may affect cancer progression through different phosphorylation sites or N^6^‐methyladenosine site modifications.
3. BRD9 could be used as a novel biomarker for diagnosis of multiple cancer types.
4. Our functional study of BRD9 showed interaction with SMARCD1. Knockdown of BRD9 could inhibit the progression of lung and colon cancers through the Wnt/β‐catenin signalling pathway.

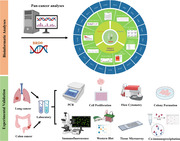

## 1 INTRODUCTION

SWItch/Sucrose Non‐Fermentable (SWI/SNF) constitutes a multi‐protein complex tasked with the regulation of gene expression in eukaryotes. It is found to remodel chromosomes mainly by destroying nucleosomes. SWI/SNF is shown to be mutated in a multiplicity of malignant tumours as well as being associated with the survival time of cancer patients.^1^ SWI/SNF includes three categories: canonical BRG1/BRM‐associated factor (cBAF), non‐canonical BAF (ncBAF) and polybromo‐associated BAF (PBAF).^2^ Bromodomain‐containing protein 9 (*BRD9*) is a recently discovered protein coding gene and is a unique member of the ncBAF complex.^3^ *BRD9* includes two domains: a DUF3512 domain and a bromodomain,^4^ the latter being the source of the protein's ability to mediate epigenetic modification. Specifically, the bromodomain of *BRD9* regulates gene transcription by recruiting the ncBAF complex to the promoter.^5^

According to the results of genomic sequencing, mutations in one or more genes responsible for encoding subunits within the SWI/SNF chromatin‐remodelling complexes are found in approximately 25% of cancer patients. In contrast to the extensively studied tumour suppressor genes and oncogenes, which have been the focus of research for decades, the impact of mutations in SWI/SNF genes on cancer remains inadequately understood. In recent years, these genes have gained more rapport, with an increasing number of studies dedicated to exploring the potential therapeutic significance of mutations in genes that encode SWI/SNF subunits. For example, many researchers have focused on their mechanism of promoting cancer and promising targeted therapies.^6^ There have been some studies showing that BRD9 as a target for anticancer drugs has potential clinical value in some specific cancer types. Specifically, *BRD9* bromodomain inhibitors hinder the proliferation of cancer cells while inducing apoptosis in acute myeloid leukaemia,^4^, ^7^ rhabdoid tumour^8^ and triple‐negative breast cancer.^9^

**FIGURE 1:**
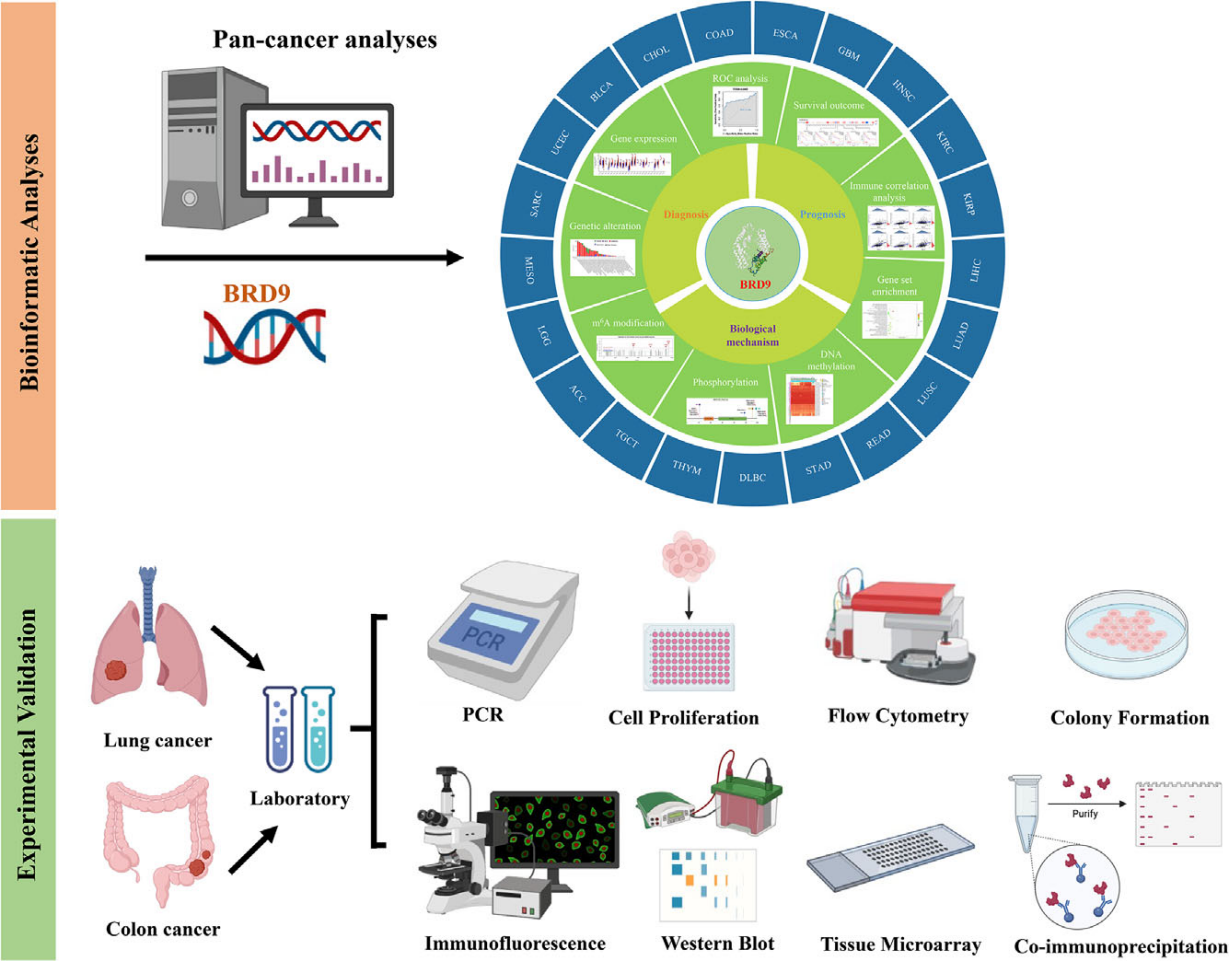
A workflow of this study. The batch‐corrected, normalised expression data and clinical information of The Cancer Genome Atlas (TCGA) pan‐cancer and target datasets were retrieved. The comprehensive pan‐cancer analysis of BRD9 included gene expression, protein expression, survival status, mutation, protein phosphorylation, DNA methylation, N^6^‐methyladenosine (m^6^A) modification, gene set enrichment analysis, receiver operating characteristic (ROC) analysis, immune correlation analysis, a least absolute shrinkage and selection operator (LASSO) regression analysis, etc. For experimental validation, tissue microarrays from lung and colon cancer patients were used to detect the expression of bromodomain containing 9 (BRD9). The biological functions of *BRD9* and the signalling pathway involved have also been explored in lung cancer and colorectal cancer cell lines. The experiments contained PCR, cell proliferation, flow cytometry, colony formation, immunofluorescence, co‐immunoprecipitation and Western blot.

Although some existing evidence based on cytological or in vivo experiments supported the association between *BRD9* and some specific cancers, there has been no reported comprehensive analysis for *BRD9* in pan‐cancer. Performing a comprehensive analysis of *BRD9* with potential clinical value and evaluation of its association with prognosis and potential molecular mechanisms can offer fresh insights into our understanding of tumourigenesis and the advancement of cancer therapies. In Figure 1, we utilised The Cancer Genome Atlas (TCGA) database to conduct an inaugural pan‐cancer analysis of *BRD9*, encompassing but not limited to gene expression, protein expression, survival status, mutation, protein phosphorylation, DNA methylation, N^6^‐methyladenosine (m^6^A) modification, gene set enrichment analysis, immune correlation analysis, etc. We followed up the bioinformatics analysis with experimental validation, employing tissue microarrays from lung and colon cancer patients to detect the expression of BRD9. The biological functions of *BRD9* and the signalling pathway involved were also explored in cell lines related to lung cancer and colon cancer.

## 2 MATERIAL AND METHODS

### 2.1 BRD9 expression profile data analysis

Along with corresponding clinical information, raw RNA‐seq data counts were acquired from the TCGA portal. The raw data were then processed by converting counts to transcripts per million (TPM) and normalising it using log2(TPM + 1). Tissue samples containing associated clinical information were retained, resulting in a dataset of these samples for subsequent analysis.

*BRD9* expression was compared between tumour and their paired adjacent normal tissues across various tumour types present in the TCGA database. The statistical differentiation was assessed by Wilcoxon signed‐rank test. The TCGA cancer types lacking corresponding normal sample were excluded from this analysis.

To supplement the data from TCGA database, we also utilised a proteomic database—Clinical Proteomic Tumor Analysis Consortium (CPTAC)^10^ to access BRD9's protein expression. The advantage of validation at the protein level is that while most other databases analyse gene expression at the mRNA level, CPTAC can more accurately reflect the physiologic state of the disease.

Violin plots based on *BRD9* expression in different pathological stages were revealed via the ‘Expression analysis‐stage Plot’ module of Gene Expression Profiling Interactive Analysis (GEPIA2, version 2) online tool (http://gepia2.cancer‐pku.cn/#analysis).^11^

The spatial transcriptome analysis for BRD9 was performed based on SpatialDB database (http://www.spatialomics.org/SpatialDB/).^12^

### 2.2 The human protein atlas

Data on BRD9 expression in cancer cell lines and cancer tissues were obtained from human protein atlas (HPA) (https://www.proteinatlas.org).^13^

### 2.3 Survival analysis

Overall survival (OS) and disease‐free survival (DFS) of *BRD9* across all TCGA cohorts were analysed using ‘Survival Analysis’ module in GEPIA2, and survival maps and plots were generated.^11^ Kaplan–Meier curves were generated via the ‘survminer’ package. For survival map, we examined the impact of *BRD9* on survival across various cancer types, employing the Mantel–Cox test for estimation and comparison. In the survival plots, cohorts with high and low *BRD9* expression levels were separated based on median values.

### 2.4 Genetic mutation analysis

We entered ‘BRD9’ in the ‘Quick Search’ section of the cBioPortal for Cancer Genomics (http://cbioportal.org).^14^, ^15^ The alteration frequency for all the tumour types represented in the TCGA database was shown using ‘Cancer Types Summary’ module. Mutation sites within BRD9 were represented in both the schematic diagram of its protein structure and the 3D structure, both of which were accessible within the ‘Mutations’ module. Kaplan–Meier curves were shown in specific TCGA cancer types, both with and without BRD9 genetic alterations, using the ‘Comparison/Survival’ module.

### 2.5 m^6^A modification analysis

A heatmap obtained from the ‘Exploration‐Gene_Corr’ panel of TIMER 2.0 was utilised to represent the correlation analysis between *BRD9* and nineteen m^6^A regulators in diverse cancer types sourced from the TCGA database. The purity‐adjusted Spearman's rank correlation test was performed to generate the partial correlation coefficient (cor) and its corresponding *p*‐value. When m^6^A regulators were mutated, *BRD9* expression was shown by the ‘Gene_Mutation’ panel of TIMER 2.0.

The prediction of m^6^A modification sites within *BRD9* was carried out using the sequence‐based RNA adenosine methylation site predictor (SRAMP) web tool (http://www.cuilab.cn/sramp/) in full transcript mode with the following settings: analyse RNA secondary structure—no; tissue—generic; and show query sequence as RNA.^16^

### 2.6 Experimental validation

Experimental validation was performed in tissue microarrays and cell lines derived from lung and colon cancers, including quantitative polymerase chain reaction (qPCR) analysis, cell proliferation assay, apoptosis analysed by flow cytometry, colony formation assay, immunofluorescence, Western blot, immunochemistry and co‐immunoprecipitation (co‐IP). The experimental set‐up was described in detail in the Supporting Information.

### 2.7 Constructing and validating the prognostic signature in TCGA cohorts

To compare and assess the predictive accuracy of each gene and risk score, timeROC analysis was conducted.^17^ Feature selection was conducted by the least absolute shrinkage and selection operator (LASSO) regression algorithm via ‘glmnet’ (version 4.1‐1) package, with a 10‐fold cross‐validation approach.^18^ Univariate Cox proportional hazard regression and log‐rank tests were performed.

### 2.8 Spearman's correlation analysis

The infiltration level of immune cells was calculated though the TIMER algorithm using the R package ‘immunedeconv’.^19^ Spearman's correlations between the immune cell infiltration levels and the risk score of the prognostic signature were calculated, and the results were compared using the ‘ggstatsplot’ package.

### 2.9 The prediction of potential drug compounds

The potential drug compounds were predicted from the Drug Signatures Database (DSigDB) accessed through the Enrichr platform (https://maayanlab.cloud/Enrichr/) according to similar differentially expressed genes.^20^

## 3 RESULTS

### 3.1 Expression profile of BRD9

**FIGURE 2:**
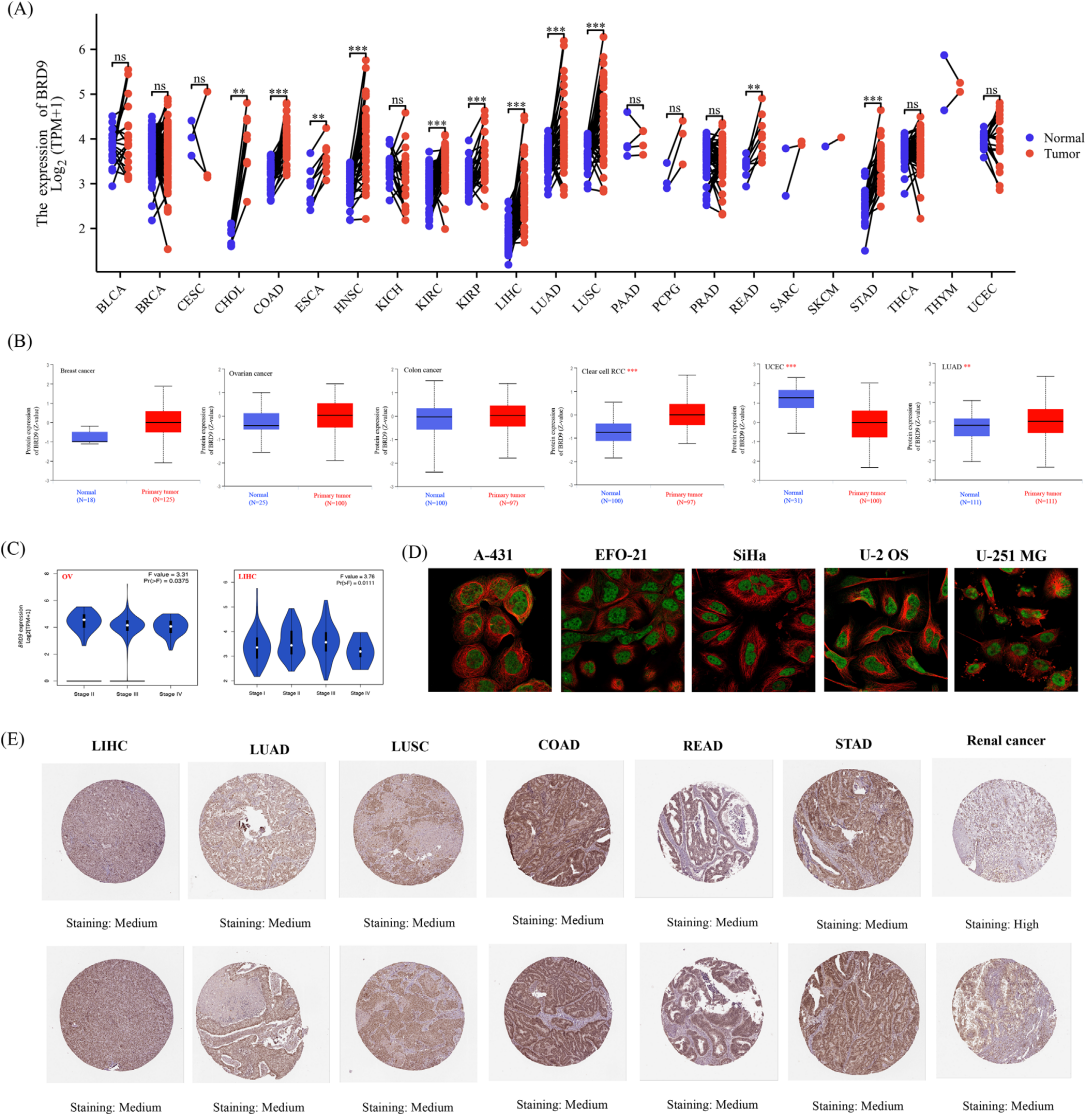
Bromodomain containing 9 (*BRD9*) expression in multiple tumours and major pathological stages. (A) Compared with paired normal samples, *BRD9* expression levels in tumour samples from multiple cancers types were obtained based on The Cancer Genome Atlas (TCGA) data. There were cases where the sample size was less than three or the within‐group standard deviation (SD) was 0 (such as sarcoma [SARC], skin cutaneous melanoma [SKCM], thymoma [THYM]), these groups were not included for statistical analysis, but visualisation was performed. No normal samples were available for some cancer types (e.g., adrenocortical carcinoma [ACC], breast invasive carcinoma [BRCA], lymphoid neoplasm diffuse large B‐cell lymphoma [DLBC], etc.). (B) At the protein level, BRD9 expression between normal tissue and primary tissue of breast cancer, ovarian cancer, colon cancer, clear cell renal cell carcinoma (RCC), uterine corpus endometrial carcinoma (UCEC) and lung adenocarcinoma (LUAD) were analysed according to data from the Clinical Proteomic Tumour Analysis Consortium (CPTAC) dataset. (C) The *BRD9* expression levels were detected according to pathological stage in ovarian serous cystadenocarcinoma (OV) and liver hepatocellular carcinoma (LIHC) (*p* < .05). Only statistically significant differences were shown. log2(TPM + 1) was calculated for log‐scale, where TPM represents transcripts per million. (D) *BRD9* expression was detected by immunofluorescence. BRD9 was labelled with green fluorescence. Microtubules were labelled with red fluorescence. (E) Representative images of BRD9 expression in cancer tissue samples were shown. There were two immunohistochemistry images for each cancer types. ^*^ *p* < .05; ^**^ *p* < .01; ^***^ *p* < .001.

In Figure 2A and Table S1, compared with their matched adjacent normal samples, *BRD9* exhibited significant upregulation in the tumour tissue across various cancer types, including cholangiocarcinoma (CHOL), colon adenocarcinoma (COAD), esophageal carcinoma (ESCA), head and neck squamous cell carcinoma (HNSC), kidney renal clear cell carcinoma (KIRC), kidney renal papillary cell carcinoma (KIRP), liver hepatocellular carcinoma (LIHC), lung adenocarcinoma (LUAD), lung squamous cell carcinoma (LUSC), rectum adenocarcinoma (READ) and stomach adenocarcinoma (STAD).

At the protein level in Figure 2B, BRD9 was significantly upregulated in clear cell renal cell carcinoma (RCC) and LUAD, while it exhibited a significantly downregulated in uterine corpus endometrial carcinoma (UCEC).

In Figure 2C, compared in different pathological stage, the *BRD9* expression showed significant difference (*p* < .05) only in ovarian serous cystadenocarcinoma (OV) and LIHC.

Immunofluorescence assay identified that BRD9 was highly expressed in most cancer cell lines, such as epidermoid carcinoma cell line A‐431, human osteosarcoma cell line U‐2 OS, human glioblastoma cell line U‐251 MG, human ovarian carcinoma cell line EFO‐21 and squamous cell carcinoma cell line SiHa (Figure 2D). Similarly, BRD9 also showed elevated expression in tissue samples from LIHC, LUAD, LUSC, COAD, READ, STAD and renal cancer (Figure 2E).

### 3.2 OS and DFS analysis

**FIGURE 3:**
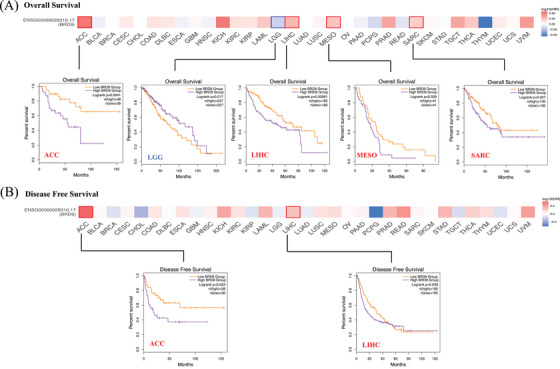
The relationship between bromodomain containing 9 (*BRD9*) expression and survival outcome of multiple cancer types in The Cancer Genome Atlas (TCGA) database. According to log10 (hazard ratio [HR]), overall survival (A) and disease‐free survival (B) for different patient cohorts were displayed on the survival maps. Kaplan–Meier curves with positive results were shown (*p* < .05).

We indicated that elevated *BRD9* expression was linked to an adverse prognosis, with regards to OS, for adrenocortical carcinoma (ACC), LIHC, mesothelioma (MESO) and sarcoma (SARC) cohorts (Figure 3A). Conversely, low expression of *BRD9* was linked to a unfavourable outcome for OS in lower grade glioma (LGG) brain cancers. Figure 3B showed elevated *BRD9* expression was associated with an unfavourable prognosis, with regards to DFS, for cancers of ACC and LIHC within the TCGA database.

### 3.3 BRD9 mutation analysis

**FIGURE 4:**
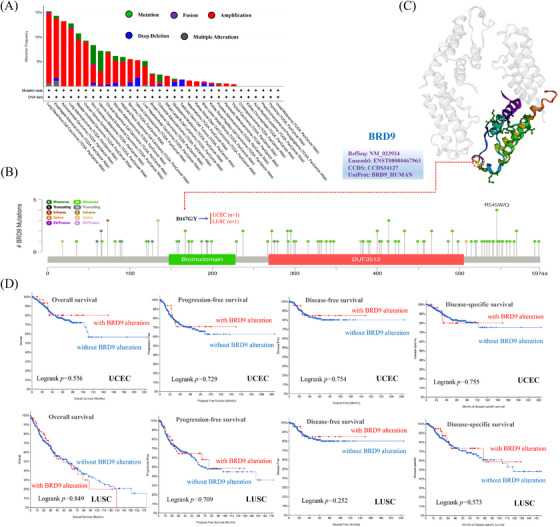
Genetic alteration of bromodomain containing 9 (BRD9) in different tumours based on The Cancer Genome Atlas (TCGA) database by using the cBioPortal tool. The alteration frequency with mutation type (A) and mutation site (B) for multiple cancers were shown. The mutation site with the highest alteration frequency (D167G/Y) in the bromodomain of BRD9 (C). The correlation between BRD9 mutation status and overall, disease‐specific, disease‐free and progression‐free survival of uterine corpus endometrial carcinoma (UCEC) and lung squamous cell carcinoma (LUSC) were analysed (D).

Figure 4A and Table S2, sourced from cBioPortal, display the genetic alteration status of BRD9 for patient samples. The highest alteration frequency of BRD9 occurs among patients with LUSC, where the primary type of alteration was ‘amplification’ referring to copy number alteration (CNA). The following six types of cancer with genetic alteration had especially high copy number amplification of BRD9: ACC, lymphoid neoplasm diffuse large B‐cell lymphoma (DLBC), KIRC, MESO, pancreatic adenocarcinoma (PAAD) and thyroid carcinoma (THCA). CNA of BRD9 was particularly pronounced in testicular germ cell tumours (TGCT), where copy number deep deletion made up all the genetic alterations to BRD9 in that particular cancer. Figure 4B,C showed the numerous mutation sites of BRD9 among the TCGA cohort, with emphasis on site with the highest alteration frequency (D167G/Y) in bromodomain in the 3D structure of BRD9. Two samples from the TCGA database were found to be mutated at this shared site: one sample from UCEC cases and another sample from LUSC cases. However, the D167G/Y alteration of BRD9 did not show any effect on the clinical survival on the two UCEC and LUSC cases (Figure 4D).

### 3.4 Tumour mutation burden/microsatellite instability analysis associated with *BRD9*

High tumour mutation burden (TMB) has been shown to be a biomarker for immunotherapeutic response for treatments that inhibit programmed cell death 1/programmed death‐ligand 1 (PD‐1/PD‐L1) ligation in multiple tumours, thereby improving the survival of cancer patients.^21^ Similarly, microsatellite instability (MSI) has been suggested as a potential marker for immune checkpoint blockade therapy.^22^ The relation between *BRD9* expression and TMB across all tumour types indicated that *BRD9* expression was positively related with TMB in ACC, LUAD, MESO, PAAD and skin cutaneous melanoma (SKCM), but negatively related with that in breast invasive carcinoma (BRCA), THCA, thymoma (THYM) and uveal melanoma (UVM) (Figure S1A). *BRD9* expression was positively related with MSI in bladder urothelial carcinoma (BLCA), LUAD, LUSC, SARC and STAD, and negatively related with that in DLBC and pheochromocytoma and paraganglioma (PCPG) (Figure S1B).

### 3.5 Phosphorylation site analysis associated with BRD9

Positive phosphorylation sites were illustrated in a schematic diagram of the BRD9 protein, comparing normal tissues to primary tumour tissues (Figure S2A), and variations in BRD9 phosphorylation levels were depicted using box plots for different cancer types (Figure S2B–F). In the LUAD cohort, no significant differences were observed in the phosphorylation levels of BRD9 between normal tissues and primary tumour tissues.

In contrast to normal tissues, the phosphorylation levels of the T103 locus, situated outside the bromodomain domain of BRD9, exhibited an increase in breast cancer and clear cell RCC tissues but a decrease in UCEC and colon cancer tissues. The phosphorylation level of the S568/S588 locus, located outside the DUF3512 domain, also exhibited an increase in breast cancer, colon cancer and ovarian cancer. Additionally, it is noteworthy that in comparison with normal tissues, the S482 locus within the DUF3512 domain of BRD9 displayed a decreased phosphorylation level in breast cancer tissues.

### 3.6 *BRD9* DNA methylation analysis

When compared with normal samples, the level of *BRD9* methylation differed significantly in 13 cancer types. We observed increased methylation levels of *BRD9* in BRCA, KIRC and KIRP (Figure S3A), and conversely reduced methylation levels in BLCA, ESCA, HNSC, LIHC, LUAD, LUSC, PAAD, prostate adenocarcinoma (PRAD), READ and UCEC (Figure S3A). Relative methylation of the eight cancer types with the most significant differences are represented as heatmaps for multiple probes in Figure S3B–I (all *p* < .0001).

### 3.7 m^6^A modification analysis associated with *BRD9*

**FIGURE 5:**
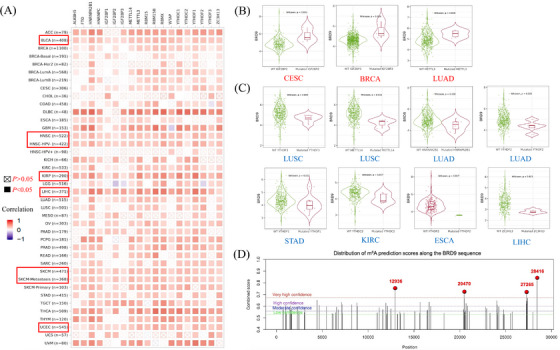
N^6^‐methyladenosine (m^6^A) modification analysis associated with bromodomain containing 9 (*BRD9*). (A) The correlation analysis between *BRD9* and 19 m^6^A regulators in different cancer types based on The Cancer Genome Atlas (TCGA) database were shown in the corresponding heatmap. (B) With mutation of any of the 3 m^6^A regulators (IGF2BP2/IGF2BP3/METTL3), *BRD9* expression was detected in different cancer types including cervical squamous cell carcinoma and endocervical adenocarcinoma (CESC), breast invasive carcinoma (BRCA) and lung adenocarcinoma (LUAD) (all *p* < .05). (C) With mutation of any of the 7 m^6^A regulators (METTL14/HNRNPA2B1/YTHDF1/YTHDF2/YTHDF3/YTHDC2/ZC3H13), *BRD9* expression was detected in different cancer types (all *p* < .05). (D) The computational identification of m^6^A modification sites were shown for *BRD9* sequence.

m^6^A, a prevalent RNA modification, plays a role in various biological processes, including tumour progression.^23^ The expression level of m^6^A regulators are intricately linked to the activity of cancer‐related signalling pathways.^24^ The heatmap in Figure 5A showedthe relationship between *BRD9* and 19 m^6^A regulators among various cancer types. Notably, *BRD9* expression was positively correlated with 19 m^6^A regulators of BLCA, HNSC, human papillomavirus (HPV)‐negative HNSC (HNSC‐HPV‐), KIRP, LIHC, SKCM, SKCM‐metastasis and UCEC. When any of the m^6^A readers (IGF2BP2 and IGF2BP3) or m^6^A writer METTL3 were mutated, *BRD9* was significantly highly expressed in cervical squamous cell carcinoma and endocervical adenocarcinoma (CESC), BRCA and LUAD (Figure 5B). In contrast, Figure 5C showed that with mutation of any of the m^6^A readers (HNRNPA2B1, YTHDF1, YTHDF2, YTHDF3 and YTHDC2) or m^6^A writers (METTL14 and ZC3H13), expression of *BRD9* was significantly downregulated in different cancer types. We also computationally identified m^6^A modification sites for the *BRD9* gene sequence (Figure 5D). The following four m^6^A sites were identified with very high confidence: 12 936, 20 470, 27 265 and 28 416. With the previous correlations in mind, this would suggest that m^6^A regulators could affect tumour progression in different cancer types by regulating *BRD9* expression.

### 3.8 *BRD9*‐related genes set enrichment analysis

*BRD9*‐related gene set enrichment analysis was conducted, and a total of 29 proteins were predicted to interacted with BRD9 (Table S3 and Figure S4A). We then integrated all tumour expression data from TCGA to determine the top 100 genes that exhibited a correlation with *BRD9* expression (Table S4). The top four genes were included. Figure S4B indicated that *BRD9* expression showed a positive correlation with that of *mediator complex subunit 10* (*MED10*) (*R* = .57), *NOP2/sun RNA methyltransferase 2* (*NSUN2*) (*R* = .65), *PAP‐associated domain containing 7* (*PAPD7*) (*R* = .61), and *thyroid hormone receptor interactor 13* (*TRIP13*) (*R* = .62). In Figure S4C, the heatmap indicated positive correlation of expression between *BRD9* and selected targeting genes (*MED10*, *NSUN2*, *PAPD7* and *TRIP13*). The intersection of *BRD9*‐binding genes and highly correlated genes showed only one common gene, *SMARCD1* (Figure S4D). Subsequently, Kyoto encyclopedia of gene and genomes pathway analysis was conducted on the genes that interacted with *BRD9* and exhibited high correlations. The findings showed that ‘axon guidance’ and ‘spliceosome’ could potentially be implicated in the influence of *BRD9* on tumour progression (Figure S4E). We additionally carried out Gene Ontology (GO) enrichment analysis on the genes that interacted with BRD9 and displayed high correlations. In biological processes analysis, they were found to be very likely be involved in the regulation of ephrin receptor signalling pathway, peptidyl–tyrosine phosphorylation and mRNA splicing via spliceosome (Figure S4F). In cellular components analysis, the genes were found to be mainly located in the nucleus and nucleoplasm. In molecular functions analysis, the genes were shown to have functions such as poly (A) RNA binding, ATP binding and protein binding.

### 3.9 Expression levels of *BRD9* in lung cancer and colon cancer from clinical samples

**FIGURE 6:**
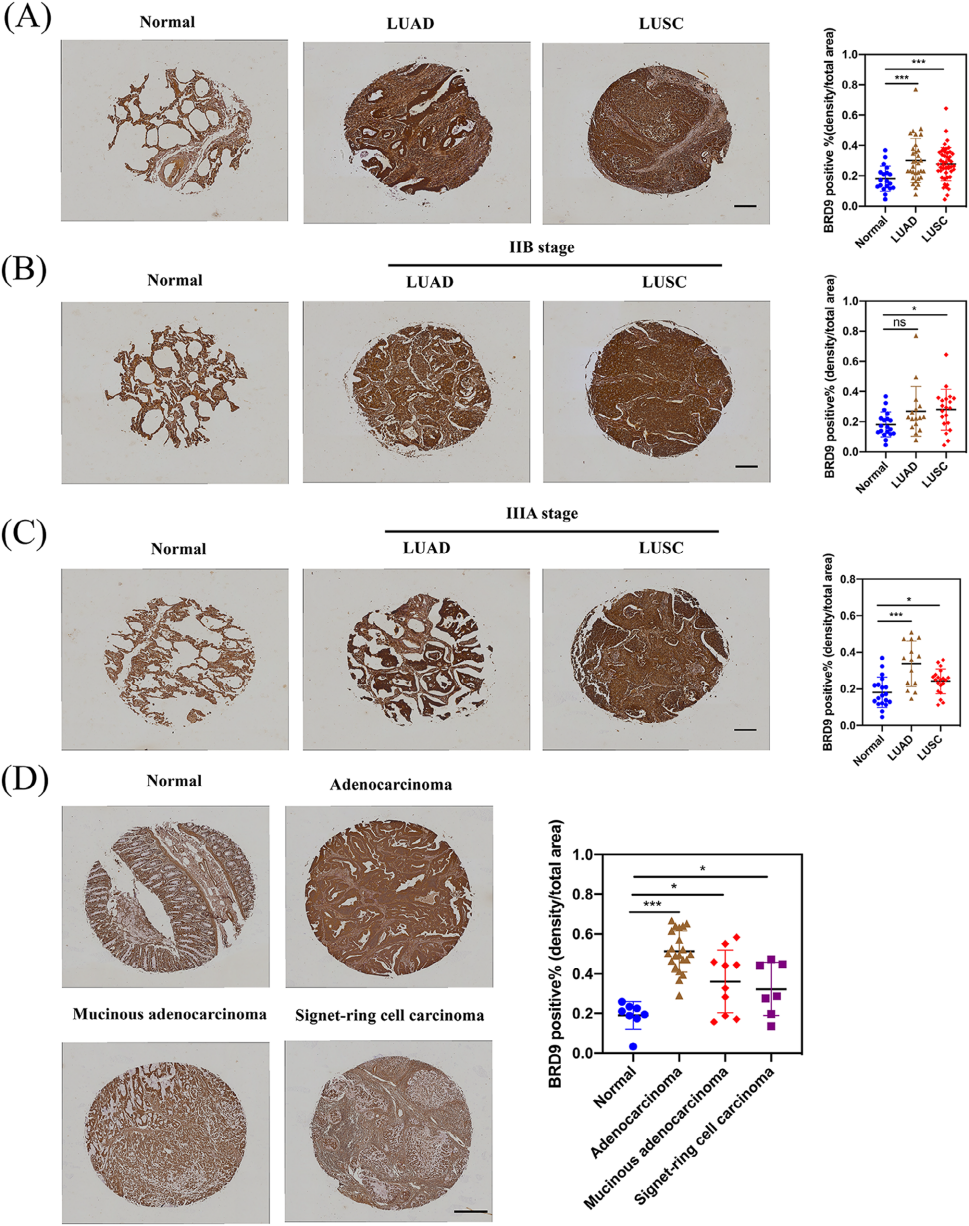
Immunohistochemical expression of bromodomain containing 9 (BRD9) in human lung cancer and colon cancer. (A) Compared with normal lung tissues, the expression of BRD9 were detected by immunohistochemistry (IHC) staining in lung cancer tissue. Especially, the expression of BRD9 were also detected in IIB stage (B) and IIIA stage (C) lung cancer tissues. Scale bar, 200 μm. (D) Compared with normal colon tissues, the expression of BRD9 were detected by IHC staining in colon cancer tissue. Scale bar, 500 μm. LUAD, lung adenocarcinoma; LUSC, lung squamous cell carcinoma; ns, not significance. ^*^ *p* < .05; ^**^ *p* < .01; ^***^ *p* < .001.

The expression of BRD9 was evaluated by immunohistochemistry. Figure 6A indicated that BRD9 staining in both LUAD and LUSC was notably higher compared to BRD9 staining in normal lung tissues. When comparing the staining of BRD9 in stage IIB lung cancers against normal lung tissues, only staining in LUSC was significantly higher (Figure 6B). Comparison of BRD9 staining between normal lung tissues and stage IIIA lung cancers once again revealed significantly higher staining in both lung cancer subtypes (Figure 6C).

Similar with lung cancer, the staining of BRD9 in colon cancer, including adenocarcinoma, mucinous adenocarcinoma and signet‐ring cell carcinoma, were also notably higher than the staining of BRD9 in normal colon tissues (Figure 6D). The patient details of tissue samples and all images taken for immunohistochemistry were shown in Figures S5 and S6.

### 3.10 The biological functions of *BRD9* in lung and colon cancers

**FIGURE 7:**
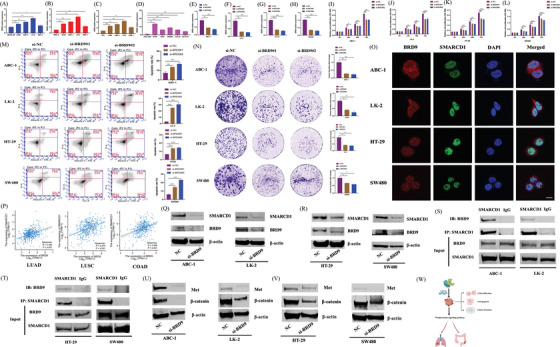
The biological functions of bromodomain containing 9 (*BRD9*) in lung and colon cancers. (A–C) The expression levels of *BRD9* were detected by quantitative real‐time PCR (qPCR) in four lung cancer cell lines compared with three different normal lung cell lines respectively. (D) The expression levels of *BRD9* were detected by qPCR in five colon cancer cell lines compared with the normal colon cell line. In ABC‐1 (E), LK‐2 (F), HT‐29 (G) and SW480 (H) cell lines, the expression level of *BRD9* was detected by qPCR after *BRD9* knockdown. The cell proliferation assay (cell counting kit‐8 [CCK‐8]) was measured in ABC‐1 (I), LK‐2 (J), HT‐29 (K) and SW480 (L) cell lines. (M) The apoptosis rate of ABC‐1, LK‐2, HT‐29 and SW480 cell lines were detected by flow cytometry. (N) A colony formation assay was performed in the four cell lines. (O) The co‐localization of BRD9 (red) and SMARCD1 (green) was detected by immunofluorescence in the four cell lines. The cells were counterstained with the nuclear probe DAPI (blue). Scale bar, 10 μm. (P) Based on The Cancer Genome Atlas (TCGA)‐lung adenocarcinoma (LUAD), lung squamous cell carcinoma (LUSC) and colon adenocarcinoma (COAD) datasets, the correlation between expression levels of *BRD9* and *SMARCD1* was analyzed. SMARCD1 proteins were detected by Western blot after BRD9 knockdown in lung cancer (Q) and colon cancer (R) cell lines. The interaction between BRD9 and SMARCD1 was detected by co‐immunoprecipitation (co‐IP) assay in lung cancer (S) and colon cancer (T) cell lines. The expression levels of key proteins in Wnt/β‐catenin signalling pathway were detected by Western blot after BRD9 knockdown in lung cancer (U) and colon cancer (V) cell lines. (W) The schematic of signalling pathway was shown. CCK‐8, cell counting kit‐8. ^*^ *p* < .05, ^**^ *p* < .01, ^***^ *p* < .001 compared with the negative control (NC).

Comparing *BRD9* expression in three different normal lung cell lines (BEAS‐2B, MRC‐9 and HLF) against each of the four different lung cancer cell lines (A549, ABC‐1, LK‐2 and EBC‐1) revealed significant upregulation of the gene in tumor. (Figure 7A–C). Among the LUAD cell lines, the highest *BRD9* expression was observed in ABC‐1 cells. Within the LUSC cell lines, LK‐2 cells exhibited the highest *BRD9* expression. Similarly, comparing the expression of *BRD9* between normal colon cells (CRL1459) and five different colon cancer cell lines revealed that *BRD9* was upregulated in colon cancer cells (Figure 7D). In COAD cell lines, *BRD9* expression was the highest in HT‐29 and SW480 cell lines. The four cell lines with the highest expression of *BRD9* in each subtype (ABC‐1, LK‐2, HT‐29 and SW480) were then selected for subsequent experiments.

qPCR was performed on the four different cells, which were transfected with siRNAs, resulting in *BRD9* knockdown (Figure 7E–H). The cell proliferation was detected, and it found that *BRD9* knockdown significantly inhibited the proliferation of ABC‐1 and LK‐2 cells (Figure 7I,J). *BRD9* knockdown also suppressed the proliferation of HT‐29 and SW480 cells (Figure 7K,L). According to the results of flow cytometry (Figure 7M), knockdown of *BRD9* promoted apoptosis in lung cancer cells (ABC‐1 and LK‐2) and colon cancer cells (HT‐29 and SW480). *BRD9* knockdown also resulted in the inhibition of colony formation in lung cancer cells and colon cancer cells (Figure 7N).

To further validate the unique association between BRD9 and SMARCD1, as shown in Figure S4D, we explored the localization of BRD9 and SMARCD1 with immunofluorescence (Figure 7O). We observed a colocalization between BRD9 and SMARCD1, where SMARCD1 was found to be in the nucleus, which was also the predominant location of BRD9. Analysis of the TCGA‐LUAD, LUSC and COAD datasets, further support this association *BRD9* expression was positively correlated with *SMARCD1* expression (Figure 7P). The Western blot results we obtained lend additional credence to this association (Figure 7Q,R), whereby BRD9 knockdown resulted in reduced SMARCD1 expression in four different cell lines. co‐IP assay also confirmed that BRD9 can interact with SMARCD1 (Figure 7S,T). Taken together, our experimental validations paint a detailed picture of the interaction between BRD9 and SMARCD1.

Incidentally, it was found that besides affecting SMARCD1 expression, BRD9 knockdown also reduced the expression levels of Met and β‐catenin in Wnt/β‐catenin signalling pathway (Figure 7U,V).

In summary, BRD9 interacts with SMARCD1. Knockdown of *BRD9* can inhibit the progression of lung and colon cancers through Wnt/β‐catenin signalling pathway (Figure 7W).

### 3.11 Potential clinical value of *BRD9*

#### 3.11.1 *BRD9* could potentially function as a novel biomarker for diagnosis across multiple tissue types

The potential diagnostic value of *BRD9* in multiple cancers was assessed through receiver operating characteristic (ROC) curve analysis, with the area under the curve (AUC) serving as the evaluation metric (Table 1 and Figure S7). Based on results from Figure 2A, six types of cancer shown significantly differential expression from TCGA pan‐cancer analysis including colorectal adenocarcinoma, ESCA, LUAD, KIRC, LIHC and STAD were selected for validation through logistic regression model. Expression profile of *BRD9* in each TCGA cohort was treated as training set, and microarray dataset from Gene Expression Omnibus(GEO) corresponding to each cancer type was used as independent validation. It showed the results of ROC analysis across multiple cancer types. Based on the TCGA cohorts, it is noteworthy that  the AUC of *BRD9* exceed .7 for several cancer types, including COAD/READ (.832), ESCA (.885), LUAD (.785), KIRC (.794), LIHC (.96) and STAD (.935) (Figure S7). Compared with TCGA cohorts, ROC curve analysis based on GEO validation cohorts of corresponding cancer types yielded similar AUC values. Moreover, the data based on confusion matrices under the optimal ‘cutoff’ from validation set of various cancer types also showed the *BRD9*‐based models can effectively discriminate between adjacent normal samples and cases of malignancy with high accuracy in these six cancer types. Although there were no GEO datasets available for KIRP and LUSC that can be used for validation, higher AUC values of these two cancer types based on the BRD9 expression profile form TCGA cohorts were also identified (Figure S8): KIRP (.769) and LUSC (.839). These results implied that *BRD9* may have the potential to be a novel biomarker for diagnosis across multiple tissue types.

**TABLE 1:** Logistic regression model for independent validation of bromodomain containing 9 (BRD9) diagnostic ability.

	TCGA (training set)	GEO (validation set)
A		
Dataset	TCGA‐COAD/READ	GSE110225−GPL96
Sample details	51 normal controls, 367 tumors	13 normal controls, 13 primary colorectal adenocarcinomas
AUC value	.832	.828
B		
Dataset	TCGA‐ESCA	GSE161533
Sample details	13 normal controls, 182 tumors	28 normal controls, 28 esophageal squamous cell carcinomas
AUC value	.885	.838
C		
Dataset	TCGA‐LUAD	GSE32863
Sample details	59 normal controls, 483 tumors	58 normal controls, 58 lung adenocarcinomas
AUC value	.785	.862
D		
Dataset	TCGA‐KIRC	GSE53757
Sample details	72 normal controls, 523 tumors	72 normal controls, 72 clear cell renal cell carcinomas
AUC value	.794	.867
E		
Dataset	TCGA‐LIHC	GSE84402
Sample details	50 normal controls, 369 tumors	14 normal controls, 14 hepatocellular carcinomas
AUC value	.96	.776
F		
Dataset	TCGA‐STAD	GSE118916
Sample details	36 normal controls, 408 tumors	15 normal controls, 15 gastric tumors
AUC value	.935	.8

Abbreviations: AUC, area under the curve; COAD, colon adenocarcinoma; ESCA, esophageal carcinoma; KIRC, kidney renal clear cell carcinoma; LIHC, liver hepatocellular carcinoma; LUAD, lung adenocarcinoma; READ, rectum adenocarcinoma; STAD, stomach adenocarcinoma; TCGA, The Cancer Genome Atlas.

#### 3.11.2 *BRD9* expression is associated with the abundance of cancer‐associated fibroblasts and the expression of immune checkpoint‐related genes

Immune response is regarded as one of the hallmarks of cancer,^25^ and immune infiltration has gradually attracted widespread attention as a prognostic factor.^26^ Cancer‐associated fibroblasts (CAFs), as the most abundant tumour‐associated stromal cells in tumour microenvironment (TME), have the capacity to impact cancer progression by regulating the functions of various tumour‐infiltrating immune cells.^27^

We employed EPIC, MCPCOUNTER, XCELL and TIDE algorithms to investigate the potential link between the prevalence of CAFs and *BRD9* expression in various cancer types within the TCGA database. We observed a positive correlation between *BRD9* expression and the estimated number of CAFs in TCGA tumours of COAD, HNSC and HNSC‐HPV−, but conversely, a negative correlation was observed in LGG (Figure S9A).

As the important role of CAFs in cancer biology has become clarified, it has gradually received more widespread attention. Targeting CAFs or their secretome can reduce immunosuppression and remodelling of the TME, which provides effective inroads for treatment of cancer. Checkpoint blockade immunotherapy enhances anti‐tumour immune response by targeting T cells. Therefore, combined checkpoint blockade immunotherapy and CAF‐targeted therapy is a strategy to treat tumours that thrive in fibroblast‐rich TMEs.^28^ SIGLEC15, TIGIT, CD274, HAVCR2, PDCD1, CTLA4, LAG3 and PDCD1LG2 are immune cell‐expressed ligands related to immune checkpoint function.^29^ The correlations between *BRD9* expression and the expression of these immune checkpoint‐related genes were analysed for 33 cancer types in TCGA database (Figure S9B). We found that the expression of all eight immune checkpoint related genes above was positively correlated with that of *BRD9* in PRAD and PCPG. However, a negative correlation was observed between BRD9 expression and the expression of all eight immune checkpoint‐related genes in TGCT.

#### 3.11.3 BRD9 has the potential to serve as a promising therapeutic target in patients with melanoma

Preclinical research on BRD9 is constantly advancing. Of all skin‐related cancers, melanoma has the highest mortality rate. Unfortunately, immunotherapies or BRAF/MEK‐based targeted therapies are only effective for a subset of melanoma patients.

**FIGURE 8:**
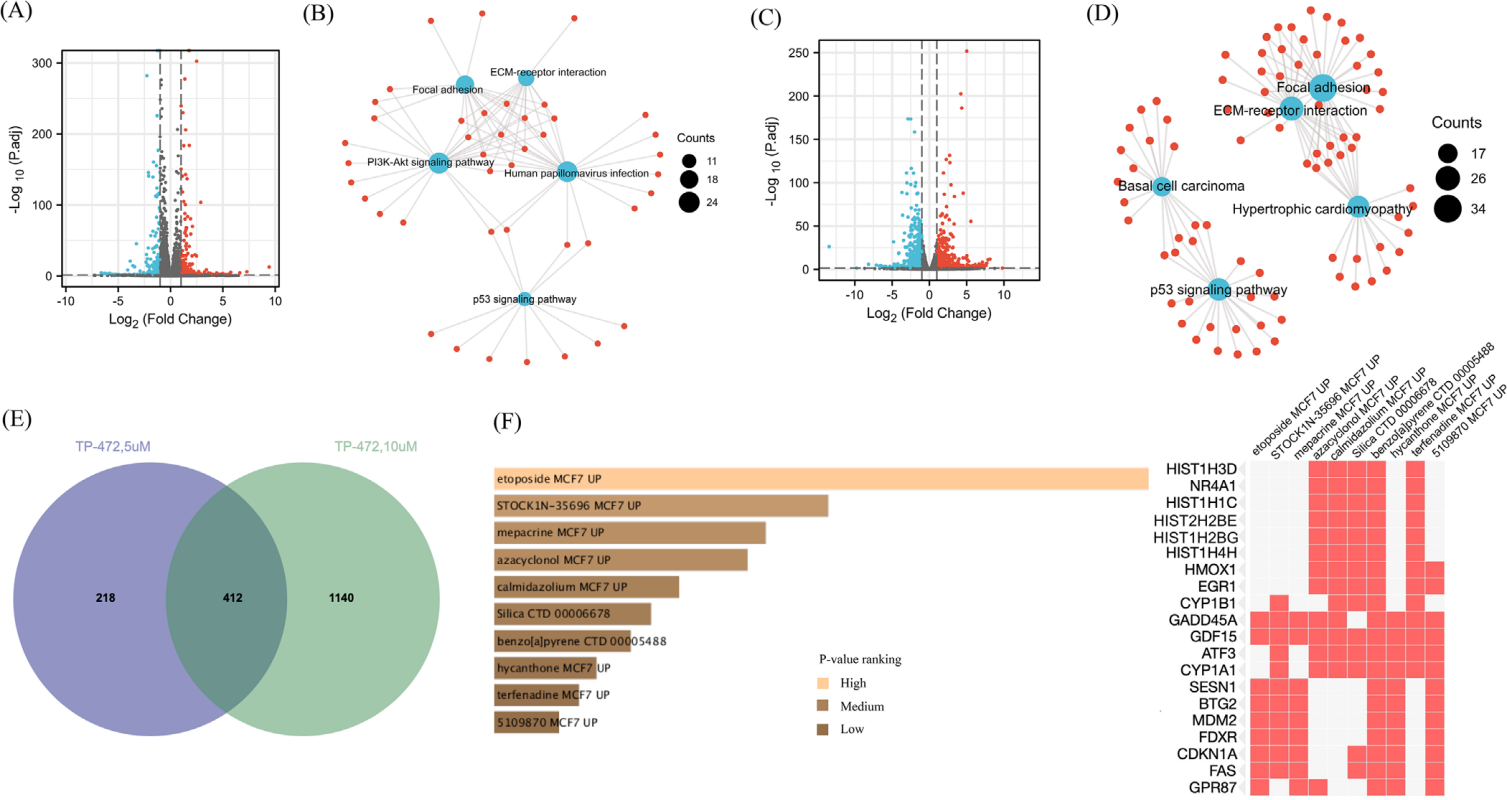
Identification of suggested top drug compounds for the similar differentially expressed genes. (A and C) These significant differentially expressed genes were obtained in volcano plot under treatment with TP‐472 at either 5 or 10 μM on A375 melanoma cells for 24 h (log_2_|FC| > 1, *p* < .05). (B and D) The Kyoto encyclopedia of gene and genomes (KEGG) analysis were conducted based on the differentially expressed genes from (A) and (C), respectively. (E) The similar differentially expressed genes was obtained between treatment with TP‐472 at 5 and 10 μM on A375 melanoma cells for 24 h. (F) The suggested top drug compounds for the similar differentially expressed genes were predicted.

A study has shown that TP‐472 as a BRD9/7 inhibitor can inhibit melanoma tumour growth by promoting apoptosis.^30^ We downloaded GSE179079, the dataset from this study, to conduct in‐depth meta‐analysis of their results. Compared with control, 342 genes significantly upregulated and 288 genes significantly downregulated were found on A375 cells treated with 5 μM TP‐472 for 24 h (Figure 8A). In addition to the previously reported findings, BRD9/7 inhibitor is very likely to inhibit the progression of melanoma by regulating p53, PI3K‐Akt or adhesion‐related signalling pathways (Figure 8B). Similarly, we identified 756 significantly upregulated genes and 796 significantly downregulated genes on A375 cells treated with 10 μM TP‐472 for 24 h (Figure 8C). Figure 8D showed that these significant differentially expressed genes may be implicated in the regulation of p53 or adhesion‐related signalling pathways.

As shown in the Venn diagram in Figure 8E, there was an overlap of 412 differentially expressed genes between Figure 8A,C. Enrichr platform was used to identify candidate drugs for these 412 differentially expressed genes. The results from the candidate drugs were generated based on *p*‐value ranking from the DSigDB database. According to the analysis results, etoposide is the prominent drug compound with which a significant number of genes were connected (Figure 8F). In a previous clinical trial, the combination of etoposide and cisplatin lacked sufficient clinical efficacy in the treatment of metastatic melanoma.^31^ Our findings would indicate that pairing etoposide with the BRD9/7 inhibitor TP‐472 would likely improve clinical outcomes in patients with melanoma.

#### 3.11.4 *BRD9* could predict the prognosis of melanoma patients undergoing anti‐PD‐1 immunotherapy

Myeloid‐derived suppressor cells (MDSCs) have the capacity to dampen anti‐tumour immune responses, facilitate the development of metastases, and led to resistance against immunotherapy.^32^ PD‐1 has been confirmed to be highly expressed in tumour‐infiltrating MDSCs.^33^ A preclinical study has shown that drug molecule targeting MDSCs enhanced the effectiveness of PD‐1 blockade in melanoma, thereby augmenting anti‐tumour activity.^34^

**FIGURE 9:**
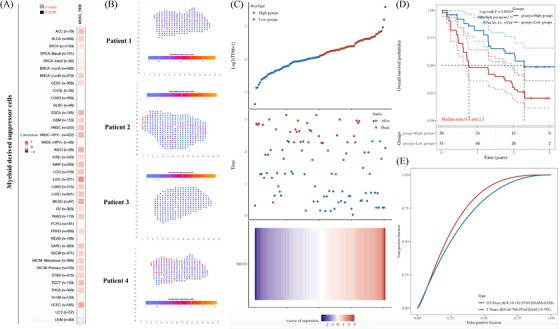
The potential of combination therapy of anti‐bromodomain containing 9 (BRD9) and anti‐programmed cell death 1 (PD‐1) in melanoma. (A) The heatmap showed a correlation analysis between *BRD9* expression and the estimated abundance of myeloid‐derived suppressor cells based on TIDE algorithm. (B) Based on spatial transcriptome data from SpatialDB database,^12^ the expression levels of BRD9 were detected in tissues of melanoma patients. (C) Distribution of risk score, overall survival (OS), OS status and heatmap of the prognostic signatures of *BRD9* in the melanoma cohort form GSE91061 dataset. (D) Kaplan–Meier curves of OS for patients with melanoma who received anti‐PD‐1 immunotherapy based on expression level of *BRD9* in GSE91061 dataset. (E) The prognostic signature was shown by the time‐dependent receiver operating characteristic (ROC) curve for predicting .5‐ and 1‐year survival based on the cohort in GSE91061 dataset. The ‘time’ in the figure uses ‘year’ as the unit. *p* < .05 was considered as statistically significant. CI, confidence interval; HR, hazard ratio.

TIDE algorithm was employed to investigate the potential connection between the predominance of MDSCs and *BRD9* expression across different cancer types within the TCGA database. A positive correlation was observed between *BRD9* expression and the estimated number of MDSCs for most cancer types including melanoma (Figure 9A). Based on spatial transcriptome data, BRD9 was shown to be highly expressed in tissues of melanoma patients (Figure 9B).

We further explored the potential of combination therapy of anti‐BRD9 and anti‐PD‐1 in melanoma, utilising data from GSE91061. Based on the median value of the prognostic risk grade, the melanoma samples were divided into low‐ and high‐risk groups. The survival of patients in the two distinct risk categories was shown in Figure 9C. The *BRD9* relative expression standards were calculated for every patient. The heatmap results revealed heightened *BRD9* expression in the high‐risk group, whereas it was comparatively lower in the low‐risk group. Survival analysis indicated that the low‐risk group exhibited a longer OS in comparison to the high‐risk group (Figure 9D). The .5‐ and 1‐year ROC curve analyses was conducted to assess the prognostic accuracy of the *BRD9* in melanoma patients undergoing anti‐PD‐1 immunotherapy. The .5‐ and 1‐year AUC values for the risk signature were .742 (95% confidence interval [CI]: .658–.826) and .708 (95% CI: .621–.795), respectively (Figure 9E).

These findings indicated that the risk score, derived from *BRD9*, has the potential to effectively forecast the prognosis of melanoma patients undergoing anti‐PD‐1 immunotherapy.

#### 3.11.5 BRD9/SMARCD1 axis may affect the immune response and predict the prognosis of LIHC and MESO patients

**FIGURE 10:**
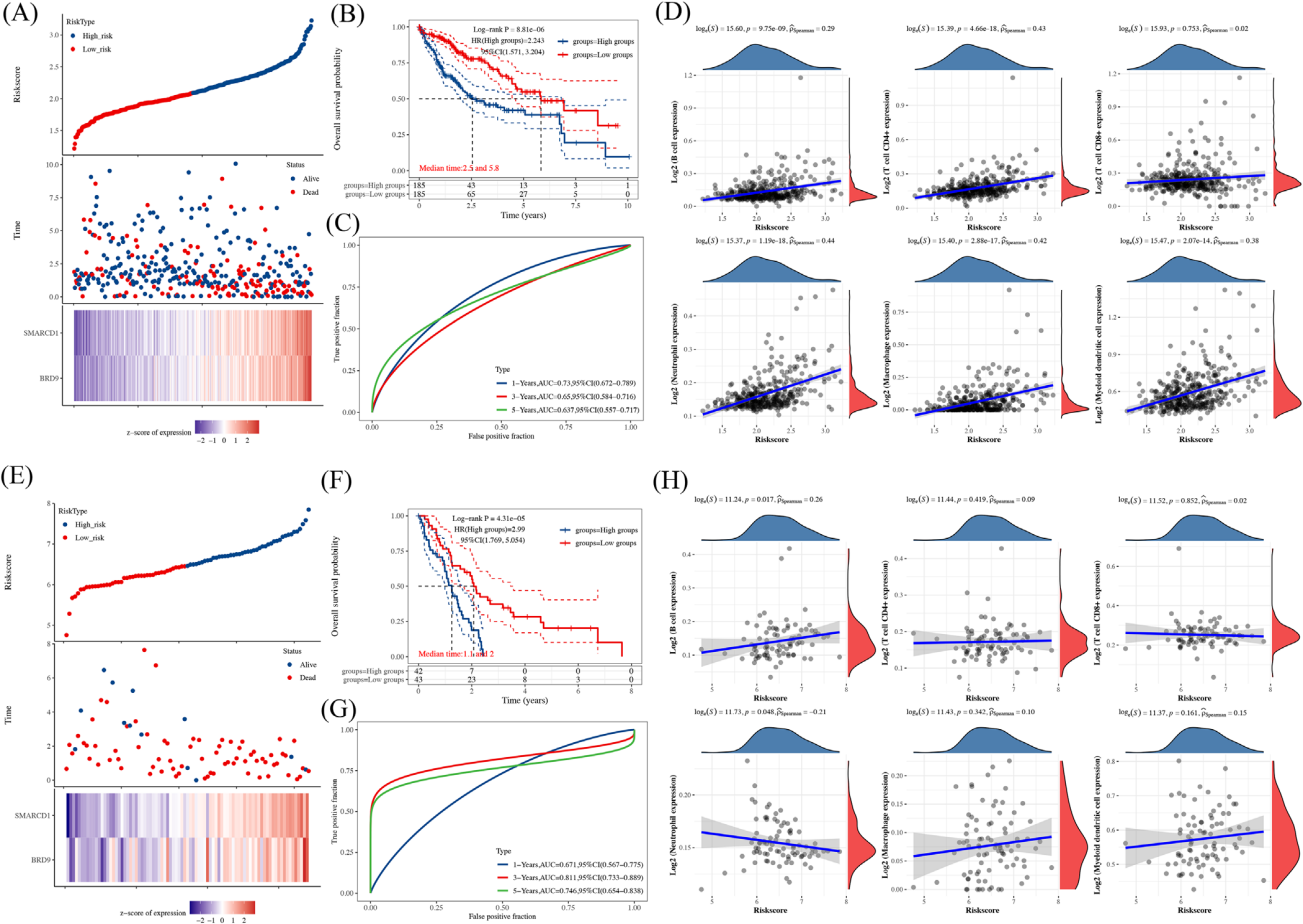
Construction and validation of prognostic signature of bromodomain containing 9 (BRD9)/SMARCD1 axis in The Cancer Genome Atlas (TCGA) cohorts and immune related Spearman's correlations analysis. (A and E) Distribution of risk score, overall survival (OS), and OS status and heatmap of the prognostic signatures of BRD9/SMARCD1 axis in the TCGA‐LIHC (A) and TCGA‐MESO cohort (E). (B and F) Kaplan–Meier curves of OS for patients with liver hepatocellular carcinoma (LIHC) based on the risk score in the TCGA cohort (A) and with mesothelioma (MESO) based on the risk score in the TCGA cohort (E). (C and G) The prognostic signature was shown by the time‐dependent receiver operating characteristic (ROC) curve for predicting 1‐, 3‐ and 5‐year survival based on TCGA‐LIHC (A) and TCGA‐MESO cohort (E). (D and H) Spearman's correlations between the infiltration level of immune cells and the risk score of the prognostic signatures were calculated based on TCGA‐LIHC (A) and TCGA‐MESO cohort (E). The ‘time’ in the figure uses ‘year’ as the unit. *p* < .05 was considered as statistically significant. CI, confidence interval; HR, hazard ratio.

As the results shown in Figure S4D, *SMARCD1* was the gene most likely to be correlated and interacted with *BRD9*. Based on the median value of the prognostic risk grade, TCGA‐LIHC samples were divided into low‐ and high‐risk groups. Figure 10A illustrated the distribution of risk grades and the survival of the two groups. The relative expression levels of *BRD9* and *SMARCD1* were calculated for each patient. The heatmap results indicated elevated expression levels of *BRD9* and *SMARCD1* in the high‐risk group, contrasted with lower expression levels observed in the low‐risk group. Survival analysis demonstrated that the low‐risk group had a longer OS compared to the high‐risk group (Figure 10B). To assess the prognostic accuracy of the BRD9/SMARCD1 axis, we conducted ROC curve analyses using the TCGA‐LIHC and TCGA‐MESO cohorts. For the TCGA‐LIHC cohort, the 1‐, 3‐ and 5‐year AUC values for the risk signature were .73 (95% CI: .672–.789), .65 (95% CI: .584–.716) and .637 (95% CI: .557–.717), respectively (Figure 10C). In the TCGA‐MESO cohort, the heatmap indicated a positive correlation between the expression levels of *BRD9* and *SMARCD1* with the risk grades (Figure 10E), and the low‐risk group exhibited a longer OS compared to the high‐risk group (Figure 10F). For the TCGA‐MESO cohort, the 1‐, 3‐ and 5‐year AUC values for the BRD9/SMARCD1 axis as prognostic signature were .671 (95% CI: .567–.775), .811 (95% CI: .733–.889) and .746 (95% CI: .654–.838), respectively (Figure 10G). We carried out a comprehensive analysis to examine the relationship between the infiltration level of immune cells and the risk score derived from the prognostic signature of BRD9/SMARCD1 axis, and found a significant positive correlation between the infiltration level of five specific immune cell types (e.g., B cells, CD4+ T cells, neutrophils, macrophage cells and myeloid dendritic cells) and the risk score associated with the prognostic signature of BRD9/SMARCD1 axis in the TCGA‐LIHC cohort (Figure 10D). According to the results of the TCGA‐MESO cohort (Figure 10H), there was a positive correlation observed between the infiltration level of B cells and the risk score associated with the prognostic signature of BRD9/SMARCD1 axis. However, the infiltration level of neutrophils exhibited a negative correlation with the risk score derived from the prognostic signature of BRD9/SMARCD1 axis. In short, the AUC values showed that the prognostic signature of BRD9/SMARCD1 axis exhibited a favourable discrimination performance in forecasting the prognosis of patients with LIHC and MESO. These findings suggested that the risk score calculated based on BRD9/SMARCD1 axis could accurately forecast the prognosis of LIHC and MESO patients. Our results also suggested that the BRD9/SMARCD1 axis might affect the immune response and could be used to predict the prognosis of LIHC and MESO patients.

## 4 DISCUSSION

Recent reports have highlighted the abnormal expression of bromodomain‐containing proteins in various cancer types. They have recently become attractive targets for drug discovery. Based on their structural domains, bromodomain‐containing proteins are classified into bromodomain and extra‐terminal (BET) and non‐BET families of bromodomain proteins. The BET family includes bromodomain containing 2, 3, 4 (BRD2, BRD3, BRD4) and bromodomain testis associated (BRDT).^35^ The results from Oncomine database^36^ showed that *BRD2*, *BRD3* and *BRD4* are highly expressed in certain cancer types (Figure S10). Although BRD4 has been recognised as a potential target for cancer therapy,^37^ none of the BRD4 inhibitors in current clinical trials have received approval from the United States Food and Drug Administration (FDA) for human use due to ‘off‐target’ effects and induced drug resistance.^37^ These drawbacks highlight a current unmet need to develop novel small molecule inhibitors for translational medical research.

A promising alternative to BRD4 inhibitors is *BRD9*‐based targeted therapy, which has made continuous progress since the protein coding gene was recently identified. We summarised the existing small molecule drugs targeting BRD9 in Table 2. For example, Crawford et al. developed GNE‐375, a small‐molecule inhibitor targeting the BRD9 bromodomain. This inhibitor has demonstrated the ability to suppress the expression of aldehyde dehydrogenase 1 family member A1 (*ALDH1A1*), which in turn prevents epigenetically defined drug resistance.^38^ Kramer et al. also found that combination therapy of BRD9 inhibitor I‐BRD9 and vincristine can be used as an effective therapeutic approach in rhabdoid tumours.^8^ Of particular note among the current repertoire of potential BRD9‐based therapies are the two BRD9 degraders: CFT8634 whose investigational new drug (IND) application was submitted^39^; and the compound FHD‐609 whose phase I study is currently recruiting (ClinicalTrials.gov Identifier: NCT04965753).^40^

**TABLE 2:** Small molecule drugs targeting bromodomain containing 9 (BRD9).

No.	Name of drug	Chemical formula	Chemical structure	Background	Reference
1	I‐BRD9	C_22_H_22_F_3_N_3_O_3_S_2_	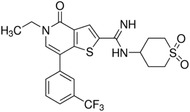	The BRD9 inhibitor was used to identify genes regulated by BRD9 in Kasumi‐1 cells involved in oncology and immune response pathways.	^41^
2	BI‐7273	C₂₀H₂₃N₃O₃	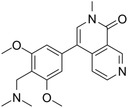	The BRD9 inhibitor was identified to mimic genetic perturbation of BRD9.	^42^
3	BI‐9564	C_20_H_23_N_3_O_3_	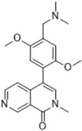	The BRD9 inhibitor displayed enhanced selectivity against the BRD7 bromodomain as well as improved pharmacokinetic properties.	^42^
4	GSK2801	C_20_H_21_NO_4_S	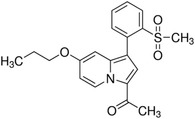	A BAZ2/BRD9 bromodomain inhibitor, synergises with BET inhibitors to induce apoptosis in triple‐negative breast cancer.	^40^
5	GSK6776	C_21_H_31_N_5_O	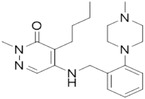	Discovery of the n‐butyl group as an atypical KAc methyl mimetic allowed generation of 31 (GSK6776) as a soluble, permeable and selective BRD7/9 inhibitor from a pyridazinone template.	^43^
6	LP99	C_26_H_30_ClN_3_O_4_S	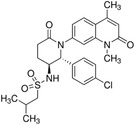	LP99 is a potent and selective bromodomain BRD9 and BRD7 inhibitor with greater potency for BRD9 compared with BRD7.	^44^
7	TP‐472	C_20_H_19_N_3_O_2_	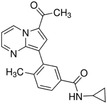	TP‐472 is a potent and selective bromodomain BRD9 and BRD7 inhibitor with greater potency for BRD9 compared with BRD7.	^45^
8	dBRD9	C_40_H_45_N_7_O_10_·2HCl	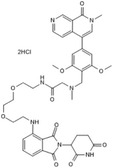	dBRD9 is a potent and selective degrader of BRD9.	^46^
9	GNE‐375	C_24_H_27_N_3_O_5_	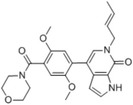	GNE‐375 is a potent and selective BRD9 inhibitor.	^43^
10	CFT8634	–	–	The investigational new drug (IND) application was submitted for the BRD9 degrader.	^39^
11	FHD‐609	–	–	The phase I study for the BRD9 degrader is currently recruiting (ClinicalTrials.gov Identifier: NCT04965753).	^40^

Although initial evaluations of the potential of *BRD9*‐based targeted therapy have been explored in their preclinical research for a small number of cancer types (Table S5), more comprehensive study of the diagnostic and prognostic potential, as well as the detailed biological mechanism of *BRD9* remain unreported. Our pan‐cancer analysis of *BRD9* provides a comprehensive framework perspective for further studies and the development of BRD9‐based targeted therapies for a variety of different diseases.

Although the non‐BET family proteins (e.g., BRD9) lack the extra‐terminal domain, they do contain one or more bromodomains. According to data from the HPA database,^47^ *BRD9* is mainly located in the nucleoplasm (Figure S11). We analysed 19 different isoforms of *BRD9* utilising the data sourced from the TCGA database. The expression level (log2(TPM + 1)) of each isoform was shown in violin plots (Figure S12). Isoform usage distribution (0%−100%) was displayed as bar plots (Figure S13).

Figure 2 suggested that the expression levels of *BRD9* were generally upregulated in most tumours when compared to their paired normal samples. Recently, some publications have provided experimental evidence that elevated expression of *BRD9* has been linked to the progression of tumours. For example, Huang et al. indicated that miR‐140‐3p directly targeted *BRD9* mRNA, leading to the inhibition of its protein translation. This resulted in decreased proliferation in LUSC by downregulating the expression of C‐myc.^48^ Similarly, Dou et al. discovered that *BRD9* promoted the progression of LIHC by activating the TUFT1/AKT pathway.^49^

Sima and coworkers analysed *BRD9* expression in multiple cancer types^50^ but produced some results that were at odds with our own findings utilising the updated TCGA database. In their publication, compared with normal tissues, *BRD9* expression was revealed to be significantly upregulated in BLCA, kidney chromophobe (KICH), THCA and UCEC, but there was no significant difference in ESCA. In our latest results, it was observed that *BRD9* was higher expressed in ESCA compared to normal tissues. However, when compared to normal tissues, no significant difference was found in *BRD9* expression in BLCA, KICH, THCA and UCEC.

The primary biological function of *BRD9* is associated with epigenetic modification, which is mediated by its bromodomain. The regulatory mechanism encompasses the recognition of acetylated lysine residues on both histones and non‐histone proteins by the bromodomain, and the recruitment of chaperones, contributing to the modulation of gene transcription.^5^ BRD9 is shown to be recruited to chromatin binding sites. While there is limited information available regarding the precise biological ligands that interact with the BRD9 bromodomain, there have been indications that BRD9 has the capacity to bind to diacetylated H4K5acK8ac and dipropionylated H4K5prK8pr.^51^ Little research has focused on *BRD9*’s other domain, DUF3512.^5^ Currently, our current knowledge is confined to the recognition that the DUF3512 domain is crucial for BRD9's interaction with other subcomplex subunits.^3^ Besides this gap in knowledge with regards to the structure of *BRD9*, there is also no evidence to support the biological significance of changes in the phosphorylation level of BRD9 protein in the occurrence and progression of cancer. Our findings in Figure S2 suggested a potential avenue for future research in this area: investigating whether S482 locus within the DUF3512 domain of *BRD9* can affect the progression of breast cancer. Following this thread of inquiry could also help reveal the diversity of function of the DUF3512 domain.

Integrating the data of Figures 2 and S3, we found that downregulation of *BRD9* DNA methylation levels in tumour samples, resulted in the upregulation of *BRD9* expression level in ESCA, HNSC, LIHC, LUAD, LUSC and READ. Conversely, this was found to be positively correlated in KIRC and KIRP. Generally, promoter hypermethylation can inhibit gene transcription.^52^ In cases of KIRC and KIRP, there was a notable negative correlation observed between *BRD9* DNA methylation and gene expression at the probes located within the promoter region. However, very few probes with significant differences (*n* = 2) were detected in these two cancer types, as shown in Figures S14 and S15. Although DNA methylation generally has the effect of inhibiting gene transcription, some publications in recent years have put forward new viewpoints. For example, research published in *Science* by Harris et al. showed that DNA methylation can activate gene expression.^53^ This could be an explanation for the results in KIRC and KIRP, but it would also suggest a more complex mechanism involved in the influence of DNA methylation on *BRD9* gene expression, which should be explore further.

Our results in Figure 4D indicated that, in cases of LUSC and UCEC, the D167G/Y alteration of BRD9 did not affect the prognosis of patients. To pursue this line of reasoning further, we explored the landscape of mutation profiles in LUSC and UCEC samples by employing the ‘maftools’ package. We analysed somatic mutation data obtained from the TCGA database for these two cancer types. Horizontal histogram plots showed known cancer genes to have the highest mutation frequency in patients with LUSC, such as *TP53* (78%), *TTN* (71%), *CSMD3* (42%), *MUC16* (39%) and *RYR2* (37%). The mutation frequency of *BRD9*, however, is only 1% (Figure S16A). Similarly, the highest mutation frequency in patients with UCEC was *PTEN* (57%), and the lowest mutation frequency was *BRD9* (3%) (Figure S16B).

BRD9 has been reported to have the desirable attribute of being stable in blood,^7^ lending itself to a role in blood profiling, which is highly desirable due to being noninvasive, easily accessible and cost‐effective.^54^ Figure S7 highlighted the potential of *BRD9* as a diagnostic marker across various cancers, opening up the possibility for using blood assays of *BRD9* as a novel detection method to improve the accuracy of cancer screening.

At present, there exists a range of approaches for treating cancer in humans, such as chemotherapy, radiotherapy, targeted therapy and surgery. These treatments, whether administered individually or in combination, are effectual but fall just short of the fabled ‘cure for cancer’. In recent years, a novel form of anti‐cancer therapy has achieved several miracles of ‘clinical cure’ for advanced cancer cases. This groundbreaking anti‐cancer therapy is immunotherapy, with the most remarkable and widely employed form being anti‐PD‐1/PD‐L1 immunotherapy.^55^ PD‐1 is primarily found on activated T cells, whereas PD‐L1 is reported to be expressed on various types of tumour cells. Blocking the interaction between PD‐1 and PD‐L1 can enhance T‐cell responses and facilitate anti‐tumour activity. Agents that block the PD‐1/PD‐L1 pathway have been documented to exhibit substantial anti‐tumour effectiveness in the treatment of cancer patients.^56^ Some PD‐1/PD‐L1 immune drugs have even been approved by FDA, such as nivolumab and pembrolizumab (for lung cancer) and Atezolizumab (for breast cancer).^57^ Despite the promising clinical results, the effectiveness of anti‐PD‐1/PD‐L1 drugs is not consistent. There is a subset of patients who do not experience benefits from anti‐PD‐1/PD‐L1 therapy, which is termed primary resistance. Additionally, some individuals who initially respond to the treatment may later experience relapse, known as acquired resistance. Combination therapy targeting the PD‐1/PD‐L1 pathway and resistance mechanisms provides the basis for improving the sensitivity of resistant patients.^58^ Given the restricted therapeutic impact of anti‐PD‐1/PD‐L1 therapy when used in isolation, there is an immediate necessity to investigate effective combination strategies to overcome resistance to anti‐PD‐1/PD‐L1 therapy, which offers valuable insights for clinical implementation. The TCGA has a few limitations. The tumour samples collected for the atlas were from untreated patients.^59^ In addition, there is no immuno‐oncology data.^60^ We therefore used the GEO dataset to explore the prognostic potential of *BRD9* in immuno‐oncology. The results from Figure 9 indicated *BRD9* could predict the prognosis of melanoma patients undergoing anti‐PD‐1 immunotherapy, which could lead to improve immunotherapy efficiency in melanoma patients.

Based on the results of Figure 10, we further evaluated whether the prognostic signature of BRD9/SMARCD1 axis can affect the immunotherapy mediated by *PD‐L1* blockade. Analysis of TCGA‐LIHC data revealed that *PD‐L1* expression was positively related with the expression levels of both *BRD9* and *SMARCD1* (Figure S17A). In contrast, TCGA‐MESO data indicated that *PD‐L1* expression did not exhibit a significant correlation with the expression levels of either of *BRD9* or *SMARCD1* (Figure S17B). This result suggested that BRD9/SMARCD1 axis could have a specific involvement in the immune response of LIHC by regulating PD‐L1 immune checkpoint.

Patients with liver cancer are frequently diagnosed at an advanced stage, resulting in an unfavourable prognosis. LIHC accounts for over 90% of liver cancer cases with the best options for treatment being chemotherapy and immunotherapy.^61^ Based on our preclinical research, use of *BRD9* as a biomarker could effectively improve the accuracy of LIHC diagnosis. Furthermore, drug development based on BRD9/SMARCD1 axis may improve the effectiveness of immunotherapy mediated by PD‐L1 blockade.

Fang et al. found that *BRD9* facilitated the progression of hepatocellular carcinoma by activating the Wnt/β‐catenin signalling pathway.^62^ According to our experimental results, *BRD9* knockdown could inhibit the progression of lung and colon cancers by the Wnt/β‐catenin signalling pathway. As we have shown, knockdown of BRD9 also reduced SMARCD1 expression. It has been reported that SMARCD1 is a subunit of the SWI/SNF complex.^63^ These findings have confirmed, quite comprehensively that BRD9 can interact with SMARCD1.

Taken together, our pan‐cancer study revealed the diagnostic and prognostic potential, as well as the biological mechanism of *BRD9* as a novel therapeutic target in human tumours for the first time. These findings will be valuable for comprehending the biological role of BRD9 in tumourigenesis and the progression of tumours. They can serve as a foundation for future research and the design of pharmacotherapies aimed at targeting BRD9 for therapeutic purposes.

## AUTHOR CONTRIBUTIONS

Yu Chen, Yuanyuan Fu and Youping Deng designed this research. Yu Chen made figures and tables. Yu Chen performed all experiments. Yu Chen wrote and edited this manuscript. Zitong Gao performed external validation of Figures S7 and S8. Yu Chen, Zitong Gao, Isam Mohd‐Ibrahim, Hua Yang, Lang Wu, Yuanyuan Fu and Youping Deng reviewed and revised this manuscript. All authors read and approved the final manuscript.

## CONFLICT OF INTEREST STATEMENT

The authors declare they have no conflicts of interest.

## ETHICS STATEMENT

Not applicable.

## Supporting information

Supporting information

Supporting information

## Data Availability

All data generated or analysed during this study are included in this published article and its Supporting Information.

## References

[1] Savas S , Skardasi G . The SWI/SNF complex subunit genes: their functions, variations, and links to risk and survival outcomes in human cancers. Crit Rev Oncol Hematol. 2018;123:114‐131. 29482773 10.1016/j.critrevonc.2018.01.009

[2] Michel BC , D'Avino AR , Cassel SH , et al. A non‐canonical SWI/SNF complex is a synthetic lethal target in cancers driven by BAF complex perturbation. Nat Cell Biol. 2018;20(12):1410‐1420. 30397315 10.1038/s41556-018-0221-1 PMC6698386

[3] Wang X , Wang S , Troisi EC , et al. BRD9 defines a SWI/SNF sub‐complex and constitutes a specific vulnerability in malignant rhabdoid tumors. Nat Commun. 2019;10(1):1881. 31015438 10.1038/s41467-019-09891-7 PMC6479050

[4] Hohmann AF , Martin LJ , Minder JL , et al. Sensitivity and engineered resistance of myeloid leukemia cells to BRD9 inhibition. Nat Chem Biol. 2016;12(9):672‐679. 27376689 10.1038/nchembio.2115 PMC4990482

[5] Zhu X , Liao Y , Tang L . Targeting BRD9 for cancer treatment: a new strategy. Onco Targets Ther. 2020;13:13191‐13200. 33380808 10.2147/OTT.S286867 PMC7769155

[6] Mittal P , Roberts CWM . The SWI/SNF complex in cancer—biology, biomarkers and therapy. Nat Rev Clin Oncol. 2020;17(7):435‐448. 32303701 10.1038/s41571-020-0357-3 PMC8723792

[7] Del Gaudio N , Di Costanzo A , Liu NQ , et al. BRD9 binds cell type‐specific chromatin regions regulating leukemic cell survival via STAT5 inhibition. Cell Death Dis. 2019;10(5):338. 31000698 10.1038/s41419-019-1570-9 PMC6472371

[8] Kramer KF , Moreno N , Fruhwald MC , Kerl K . BRD9 inhibition, alone or in combination with cytostatic compounds as a therapeutic approach in rhabdoid tumors. Int J Mol Sci. 2017;18(7):1537. 10.3390/ijms18071537 PMC5536025 28714904

[9] Bevill SM , Olivares‐Quintero JF , Sciaky N , et al. GSK2801, a BAZ2/BRD9 Bromodomain inhibitor, synergizes with BET inhibitors to induce apoptosis in triple‐negative breast cancer. Mol Cancer Res. 2019;17(7):1503‐1518. 31000582 10.1158/1541-7786.MCR-18-1121 PMC6610760

[10] Chandrashekar DS , Karthikeyan SK , Korla PK , et al. UALCAN: an update to the integrated cancer data analysis platform. Neoplasia. 2022;25:18‐27. 35078134 10.1016/j.neo.2022.01.001 PMC8788199

[11] Tang Z , Kang B , Li C , Chen T , Zhang Z . GEPIA2: an enhanced web server for large‐scale expression profiling and interactive analysis. Nucleic Acids Res. 2019;47(W1):W556‐W560. 31114875 10.1093/nar/gkz430 PMC6602440

[12] Fan Z , Chen R , Chen X . SpatialDB: a database for spatially resolved transcriptomes. Nucleic Acids Res. 2020;48(D1):D233‐D237. 31713629 10.1093/nar/gkz934 PMC7145543

[13] Asplund A , Edqvist PH , Schwenk JM , Ponten F . Antibodies for profiling the human proteome—the Human Protein Atlas as a resource for cancer research. Proteomics. 2012;12(13):2067‐2077. 22623277 10.1002/pmic.201100504

[14] Cerami E , Gao J , Dogrusoz U , et al. The cBio cancer genomics portal: an open platform for exploring multidimensional cancer genomics data. Cancer Discov. 2012;2(5):401‐404. 22588877 10.1158/2159-8290.CD-12-0095 PMC3956037

[15] Gao J , Aksoy BA , Dogrusoz U , et al. Integrative analysis of complex cancer genomics and clinical profiles using the cBioPortal. Sci Signal. 2013;6(269):pl1. 23550210 10.1126/scisignal.2004088 PMC4160307

[16] Zhou Y , Zeng P , Li YH , Zhang Z , Cui Q . SRAMP: prediction of mammalian N6‐methyladenosine (m6A) sites based on sequence‐derived features. Nucleic Acids Res. 2016;44(10):e91. 26896799 10.1093/nar/gkw104 PMC4889921

[17] Blanche P , Dartigues JF , Jacqmin‐Gadda H . Estimating and comparing time‐dependent areas under receiver operating characteristic curves for censored event times with competing risks. Stat Med. 2013;32(30):5381‐5397. 24027076 10.1002/sim.5958

[18] Tibshirani R , Bien J , Friedman J , et al. Strong rules for discarding predictors in lasso‐type problems. J R Stat Soc Series B Stat Methodol. 2012;74(2):245‐266. 25506256 10.1111/j.1467-9868.2011.01004.x PMC4262615

[19] Sturm G , Finotello F , Petitprez F , et al. Comprehensive evaluation of transcriptome‐based cell‐type quantification methods for immuno‐oncology. Bioinformatics. 2019;35(14):i436‐i445. 31510660 10.1093/bioinformatics/btz363 PMC6612828

[20] Yoo M , Shin J , Kim J , et al. DSigDB: drug signatures database for gene set analysis. Bioinformatics. 2015;31(18):3069‐3071. 25990557 10.1093/bioinformatics/btv313 PMC4668778

[21] Goodman AM , Kato S , Bazhenova L , et al. Tumor mutational burden as an independent predictor of response to immunotherapy in diverse cancers. Mol Cancer Ther. 2017;16(11):2598‐2608. 28835386 10.1158/1535-7163.MCT-17-0386 PMC5670009

[22] Hause RJ , Pritchard CC , Shendure J , Salipante SJ . Classification and characterization of microsatellite instability across 18 cancer types. Nat Med. 2016;22(11):1342‐1350. 27694933 10.1038/nm.4191

[23] Wang S , Chai P , Jia R , Jia R . Novel insights on m(6)A RNA methylation in tumorigenesis: a double‐edged sword. Mol Cancer. 2018;17(1):101. 30031372 10.1186/s12943-018-0847-4 PMC6054842

[24] Li Y , Xiao J , Bai J , et al. Molecular characterization and clinical relevance of m(6)A regulators across 33 cancer types. Mol Cancer. 2019;18(1):137. 31521193 10.1186/s12943-019-1066-3 PMC6744659

[25] Hanahan D , Weinberg RA . Hallmarks of cancer: the next generation. Cell. 2011;144(5):646‐674. 21376230 10.1016/j.cell.2011.02.013

[26] Pages F , Galon J , Dieu‐Nosjean MC , Tartour E , Sautes‐Fridman C , Fridman WH . Immune infiltration in human tumors: a prognostic factor that should not be ignored. Oncogene. 2010;29(8):1093‐1102. 19946335 10.1038/onc.2009.416

[27] Chen X , Song E . Turning foes to friends: targeting cancer‐associated fibroblasts. Nat Rev Drug Discov. 2019;18(2):99‐115. 30470818 10.1038/s41573-018-0004-1

[28] Liu T , Han C , Wang S , et al. Cancer‐associated fibroblasts: an emerging target of anti‐cancer immunotherapy. J Hematol Oncol. 2019;12(1):86. 31462327 10.1186/s13045-019-0770-1 PMC6714445

[29] Deng C , Guo H , Yan D , et al. Pancancer analysis of neurovascular‐related NRP family genes as potential prognostic biomarkers of bladder urothelial carcinoma. BioMed Res Int. 2021;2021:1‐31. 33937395 10.1155/2021/5546612 PMC8062179

[30] Mason LD , Chava S , Reddi KK , Gupta R . The BRD9/7 inhibitor TP‐472 blocks melanoma tumor growth by suppressing ECM‐mediated oncogenic signaling and inducing apoptosis. Cancers (Basel). 2021;13(21):5516. 10.3390/cancers13215516 PMC8582741 34771678

[31] Eton O , Bajorin DF , Chapman PB , Cody BV , Houghton AN . Phase II trial of cisplatin and etoposide in patients with metastatic melanoma. Invest New Drugs. 1991;9(1):101‐103. 2026478 10.1007/BF00194558

[32] Tomela K , Pietrzak B , Galus L , et al. Myeloid‐derived suppressor cells (MDSC) in melanoma patients treated with anti‐PD‐1 immunotherapy. Cells. 2023;12(5):789. 10.3390/cells12050789 PMC10000540 36899926

[33] Nam S , Lee A , Lim J , Lim JS . Analysis of the expression and regulation of PD‐1 protein on the surface of myeloid‐derived suppressor cells (MDSCs). Biomol Ther. 2019;27(1):63‐70. 10.4062/biomolther.2018.201 PMC6319546 30521746

[34] Kim SH , Li M , Trousil S , et al. Phenformin inhibits myeloid‐derived suppressor cells and enhances the anti‐tumor activity of PD‐1 blockade in melanoma. J Invest Dermatol. 2017;137(8):1740‐1748. 28433543 10.1016/j.jid.2017.03.033

[35] Jain AK , Barton MC . Bromodomain histone readers and cancer. J Mol Biol. 2017;429(13):2003‐2010. 27890782 10.1016/j.jmb.2016.11.020

[36] Rhodes DR , Kalyana‐Sundaram S , Mahavisno V , et al. Oncomine 3.0: genes, pathways, and networks in a collection of 18,000 cancer gene expression profiles. Neoplasia. 2007;9(2):166‐180. 17356713 10.1593/neo.07112 PMC1813932

[37] Lu T , Lu W , Luo C . A patent review of BRD4 inhibitors (2013–2019). Expert Opin Ther Pat. 2020;30(1):57‐81. 31815566 10.1080/13543776.2020.1702645

[38] Crawford TD , Vartanian S , Cote A , et al. Inhibition of bromodomain‐containing protein 9 for the prevention of epigenetically‐defined drug resistance. Bioorg Med Chem Lett. 2017;27(15):3534‐3541. 28606761 10.1016/j.bmcl.2017.05.063

[39] Fischer F , Alves Avelar LA , Murray L , Kurz T . Designing HDAC‐PROTACs: lessons learned so far. Future Med Chem. 2022;14(3):143‐166. 34951318 10.4155/fmc-2021-0206

[40] Bevill SM , Olivares‐Quintero JF , Sciaky N , et al. GSK2801, a BAZ2/BRD9 Bromodomain Inhibitor, Synergizes with BET Inhibitors to Induce Apoptosis in Triple‐Negative Breast Cancer. Mol Cancer Res. 2019;17(7):1503‐1518. doi: 10.1158/1541-7786.MCR-18-1121 31000582 PMC6610760

[41] Su J , Liu X , Zhang S , Yan F , Zhang Q , Chen J . Insight into selective mechanism of class of I‐BRD9 inhibitors toward BRD9 based on molecular dynamics simulations. Chem Biol Drug Des. 2019;93(2):163‐176. 30225973 10.1111/cbdd.13398

[42] Martin LJ , Koegl M , Bader G , et al. Structure‐based design of an in vivo active selective BRD9 inhibitor. J Med Chem. 2016;59(10):4462‐4475. 26914985 10.1021/acs.jmedchem.5b01865 PMC4885110

[43] Clegg MA , Bamborough P , Chung CW , et al. Application of atypical acetyl‐lysine methyl mimetics in the development of selective inhibitors of the Bromodomain‐containing protein 7 (BRD7)/Bromodomain‐containing protein 9 (BRD9) bromodomains. J Med Chem. 2020;63(11):5816‐5840. 32410449 10.1021/acs.jmedchem.0c00075

[44] Clark PGK , Vieira LCC , Tallant C , et al. LP99: discovery and synthesis of the first selective BRD7/9 Bromodomain inhibitor. Angew Chem Int Ed. 2015;54(21):6217‐6221. 10.1002/anie.201501394 PMC4449114 25864491

[45] Karim RM , Chan A , Zhu JY , Schonbrunn E . Structural basis of inhibitor selectivity in the BRD7/9 subfamily of bromodomains. J Med Chem. 2020;63(6):3227‐3237. 32091206 10.1021/acs.jmedchem.9b01980 PMC7771325

[46] Brien GL , Remillard D , Shi J , et al. Targeted degradation of BRD9 reverses oncogenic gene expression in synovial sarcoma. Elife. 2018;7:e41305. 30431433 10.7554/eLife.41305 PMC6277197

[47] Ponten F , Jirstrom K , Uhlen M . The human protein atlas—a tool for pathology. J Pathol. 2008;216(4):387‐393. 18853439 10.1002/path.2440

[48] Huang H , Wang Y , Li Q , Fei X , Ma H , Hu R . miR‐140‐3p functions as a tumor suppressor in squamous cell lung cancer by regulating BRD9. Cancer Lett. 2019;446:81‐89. 30660651 10.1016/j.canlet.2019.01.007

[49] Dou C , Sun L , Wang L , et al. Bromodomain‐containing protein 9 promotes the growth and metastasis of human hepatocellular carcinoma by activating the TUFT1/AKT pathway. Cell Death Dis. 2020;11(9):730. 32908135 10.1038/s41419-020-02943-7 PMC7481201

[50] Saladi S , Sima X , He J , et al. The genetic alteration spectrum of the SWI/SNF complex: the oncogenic roles of BRD9 and ACTL6A. PLoS One. 2019;14(9):e0222305. 31504061 10.1371/journal.pone.0222305 PMC6736241

[51] Flynn EM , Huang OW , Poy F , et al. A subset of human bromodomains recognizes butyryllysine and crotonyllysine histone peptide modifications. Structure. 2015;23(10):1801‐1814. 26365797 10.1016/j.str.2015.08.004

[52] Herman JG , Baylin SB . Gene silencing in cancer in association with promoter hypermethylation. N Engl J Med. 2003;349(21):2042‐2054. 14627790 10.1056/NEJMra023075

[53] Harris CJ , Scheibe M , Wongpalee SP , et al. A DNA methylation reader complex that enhances gene transcription. Science. 2018;362(6419):1182‐1186. 30523112 10.1126/science.aar7854 PMC6353633

[54] Chen Y , Zitello E , Guo R , Deng Y . The function of LncRNAs and their role in the prediction, diagnosis, and prognosis of lung cancer. Clin Transl Med. 2021;11(4):e367. 33931980 10.1002/ctm2.367 PMC8021541

[55] Guo L , Wei R , Lin Y , Kwok HF . Clinical and recent patents applications of PD‐1/PD‐L1 targeting immunotherapy in cancer treatment‐current progress, strategy, and future perspective. Front Immunol. 2020;11:1508. 32733486 10.3389/fimmu.2020.01508 PMC7358377

[56] Hamanishi J , Mandai M , Matsumura N , Abiko K , Baba T , Konishi I . PD‐1/PD‐L1 blockade in cancer treatment: perspectives and issues. Int J Clin Oncol. 2016;21(3):462‐473. 26899259 10.1007/s10147-016-0959-z PMC4901122

[57] Mina LA , Lim S , Bahadur SW , Firoz AT . Immunotherapy for the treatment of breast cancer: emerging new data. Breast Cancer (Dove Med Press). 2019;11:321‐328. 32099454 10.2147/BCTT.S184710 PMC6997226

[58] Wu M , Huang Q , Xie Y , et al. Improvement of the anticancer efficacy of PD‐1/PD‐L1 blockade via combination therapy and PD‐L1 regulation. J Hematol Oncol. 2022;15(1):24. 35279217 10.1186/s13045-022-01242-2 PMC8917703

[59] Kim SY , Kawaguchi T , Yan L , Young J , Qi Q , Takabe K . Clinical relevance of microRNA expressions in breast cancer validated using The Cancer Genome Atlas (TCGA). Ann Surg Oncol. 2017;24(10):2943‐2949. 28766230 10.1245/s10434-017-5984-2 PMC5839328

[60] Hu J , Cui C , Yang W , et al. Using deep learning to predict anti‐PD‐1 response in melanoma and lung cancer patients from histopathology images. Transl Oncol. 2021;14(1):100921. 33129113 10.1016/j.tranon.2020.100921 PMC7595938

[61] Anwanwan D , Singh SK , Singh S , Saikam V , Singh R . Challenges in liver cancer and possible treatment approaches. Biochim Biophys Acta Rev Cancer. 2020;1873(1):188314. 31682895 10.1016/j.bbcan.2019.188314 PMC6981221

[62] Fang D , Wang MR , Guan JL , et al. Bromodomain‐containing protein 9 promotes hepatocellular carcinoma progression via activating the Wnt/beta‐catenin signaling pathway. Exp Cell Res. 2021;406(2):112727. 34370992 10.1016/j.yexcr.2021.112727

[63] Zhou Y , Xu Q , Tao L , et al. Enhanced SMARCD1, a subunit of the SWI/SNF complex, promotes liver cancer growth through the mTOR pathway. Clin Sci. 2020;134(12):1457‐1472. 10.1042/CS20200244 32514535

