# Supplementary material for: Pan‐cancer analyses of bromodomain containing 9 as a novel therapeutic target reveals its diagnostic, prognostic potential and biological mechanism in human tumours
Source: Clin Transl Med. 2024 Feb 1;14(2):e1543. doi: 10.1002/ctm2.1543 (PMC10835192; doi:10.1002/ctm2.1543)
Supplement: Supplementary file 1 — Supporting information [file CTM2-14-e1543-s001.docx]

**Supplementary figures with legends**

**
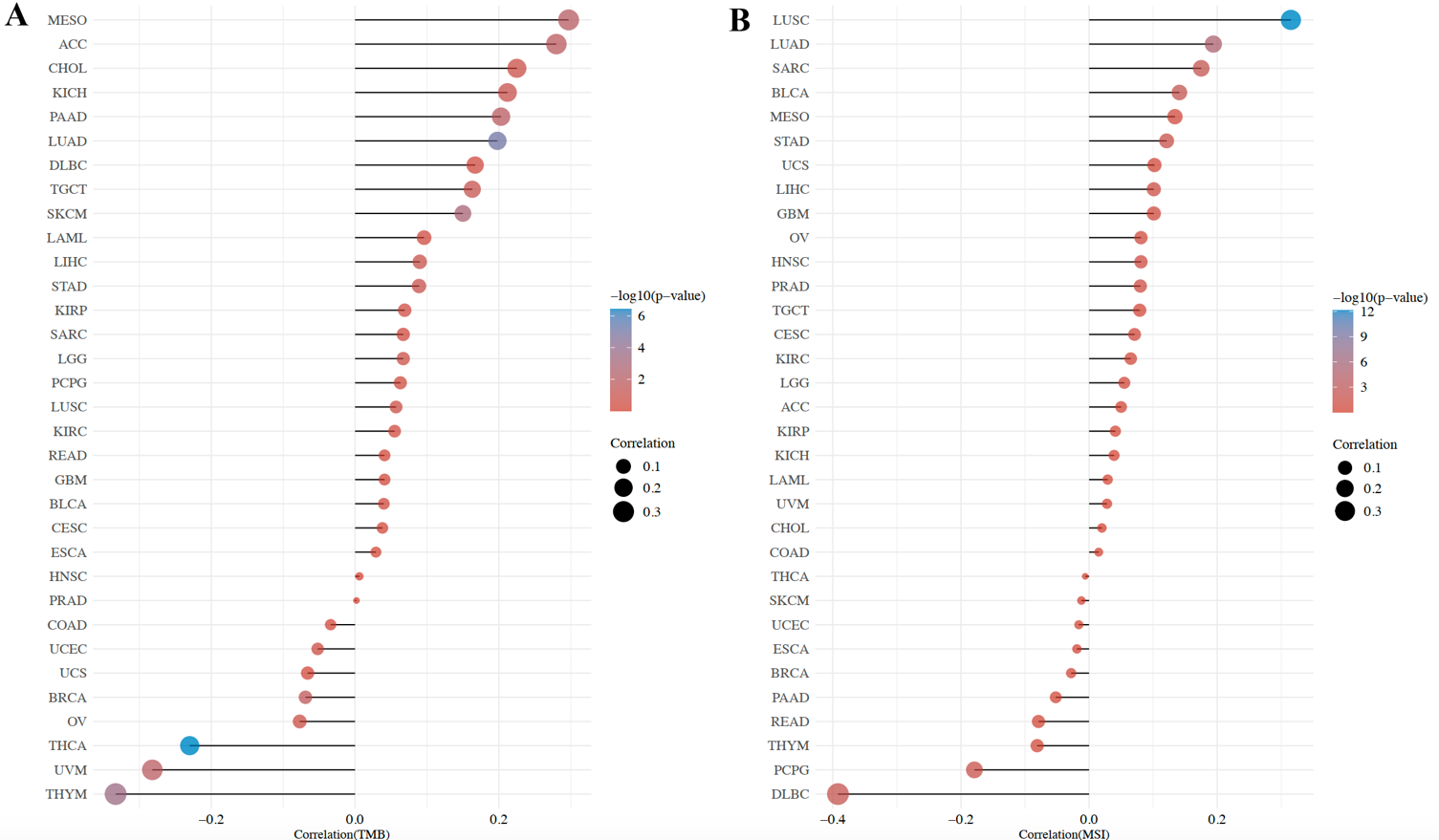
**

**Figure S1. The correlation between BRD9 expression and TMB (A)/MSI (B) across all tumors of TCGA.**

**Figure S2. Protein phosphorylation analysis of BRD9 in different tumors by using CPTAC dataset from UALCAN tool.** The schematic diagram (A) summarized the phosphoprotein sites with positive results for BRD9 (all p <0.05). BRD9 phosphoprotein expression (T103, S482, S568 and S588 sites) between normal tissue and primary tissue of breast cancer (B), ovarian cancer (C), clear cell RCC (D), colon cancer (E), UCEC (F) were analyzed (all p <0.05). Data on LUAD was not shown because there was no significant difference (p >0.05).

**
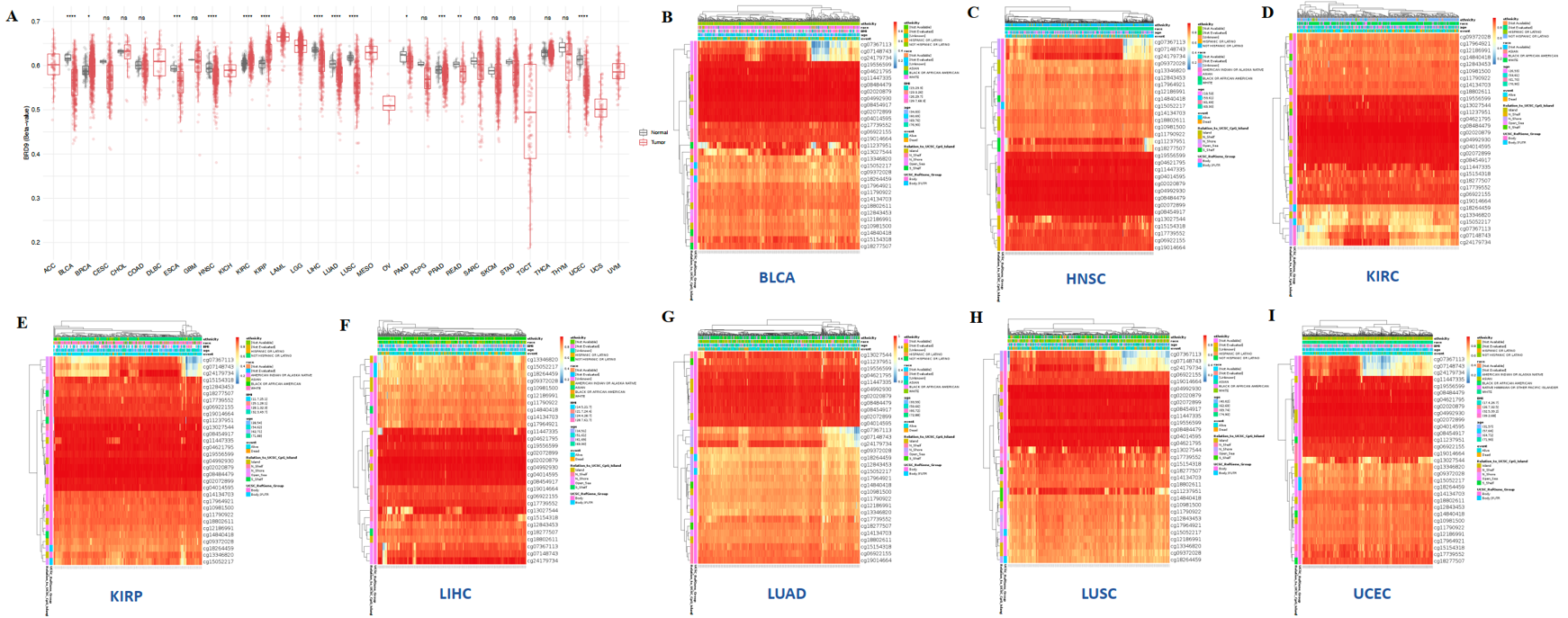
**

**Figure S3. BRD9 DNA methylation analysis in different patient cohorts from TCGA.** (A) The methylation level of BRD9 was analyzed in different tumors from the TCGA database. (B-I) The 8 cancer types with the most significant differences in (A) were selected to draw heatmaps for multiple probes (all P<0.0001). ns: not significant. * p < 0.05; ** P < 0.01; *** p < 0.001; **** p < 0.0001.

**Figure S4. BRD9-related gene set enrichment analysis.** (A) A series of BRD9-binding proteins supported by experimental evidence were obtained using the STRING tool. (B) The top 100 BRD9-correlated genes were obtained from the TCGA database. The correlation of gene expression between *BRD9* and the top 4 targeting genes, including MED10, NSUN2, PAPD7 and TRIP13, were analyzed. (C) The heatmap showed the correlation. Based on *BRD9*-binding genes and highly correlated genes, intersection analysis (D), KEGG pathway analysis (E) and GO analysis (F) were conducted.


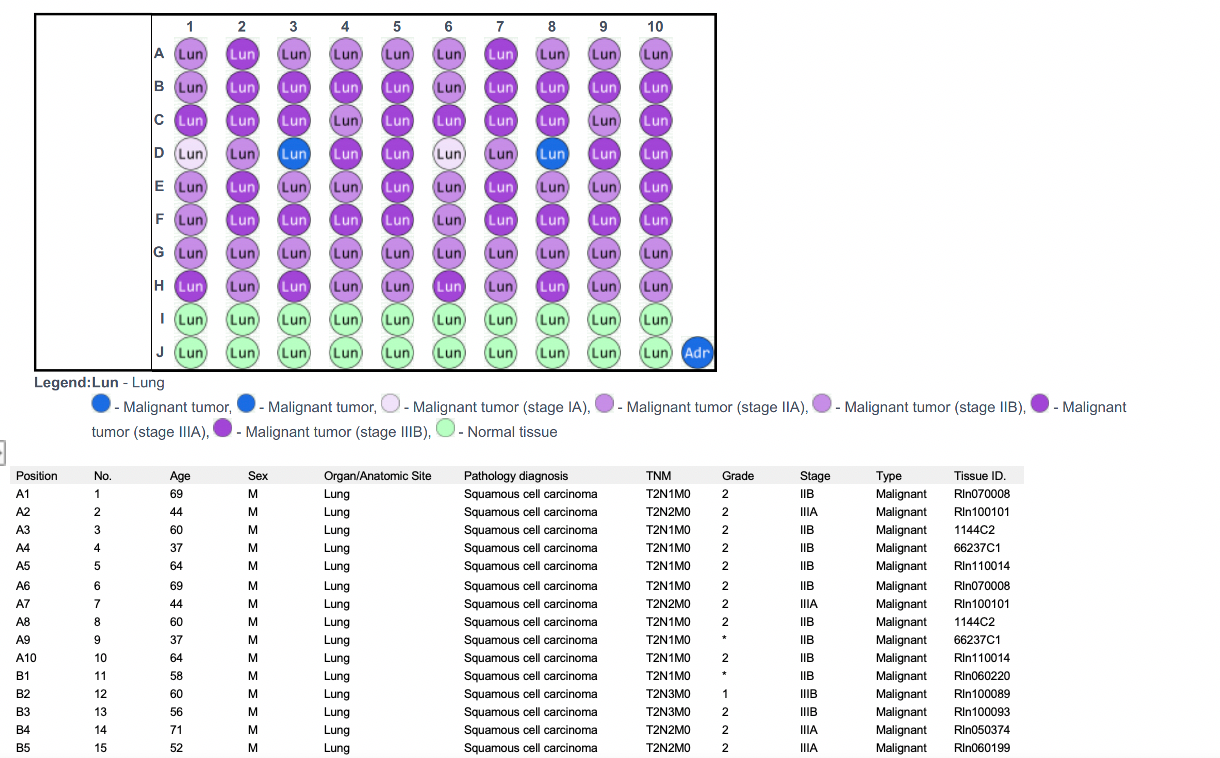


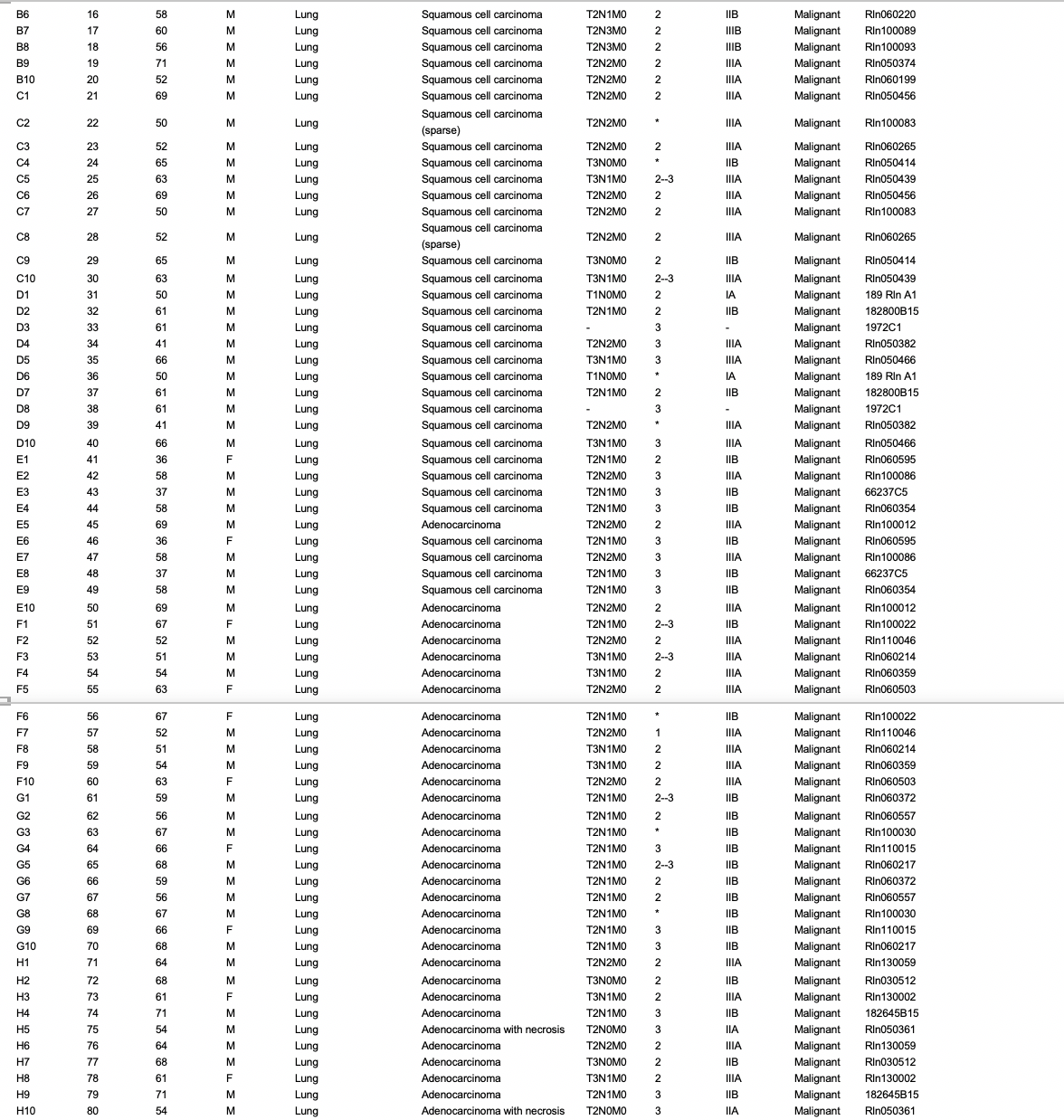


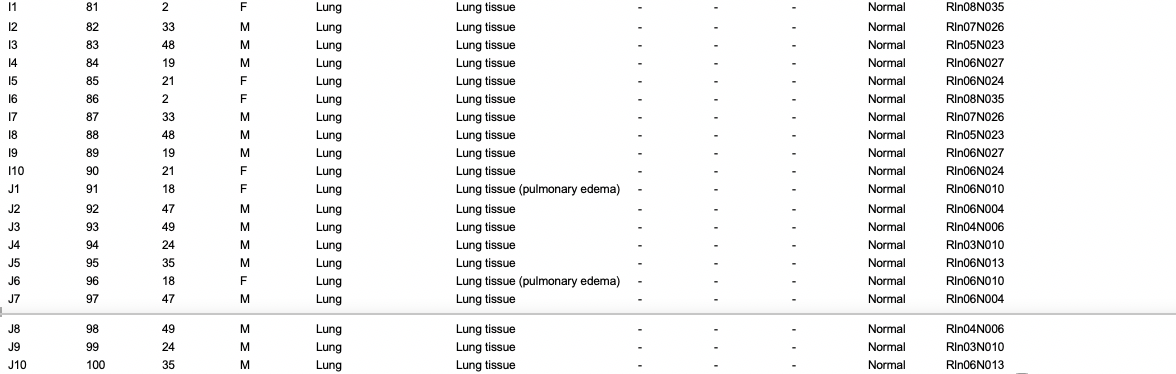


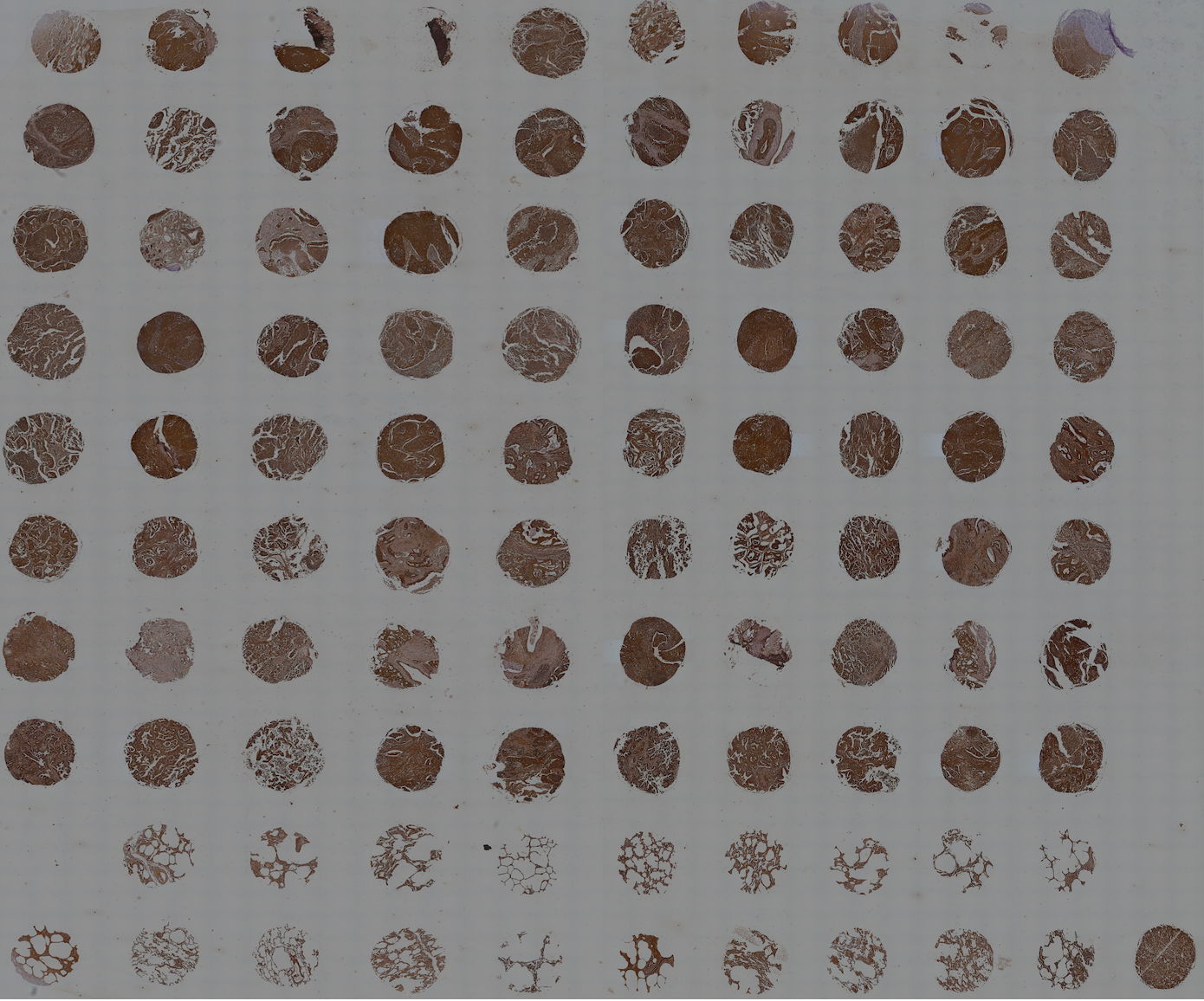


**Figure S5. The expression levels of BRD9 in lung cancer from clinical samples.** The patient details of tissue samples and all imaged taken for immunohistochemistry were included. Sample layouts were shown in the tissue microarray slide.

**
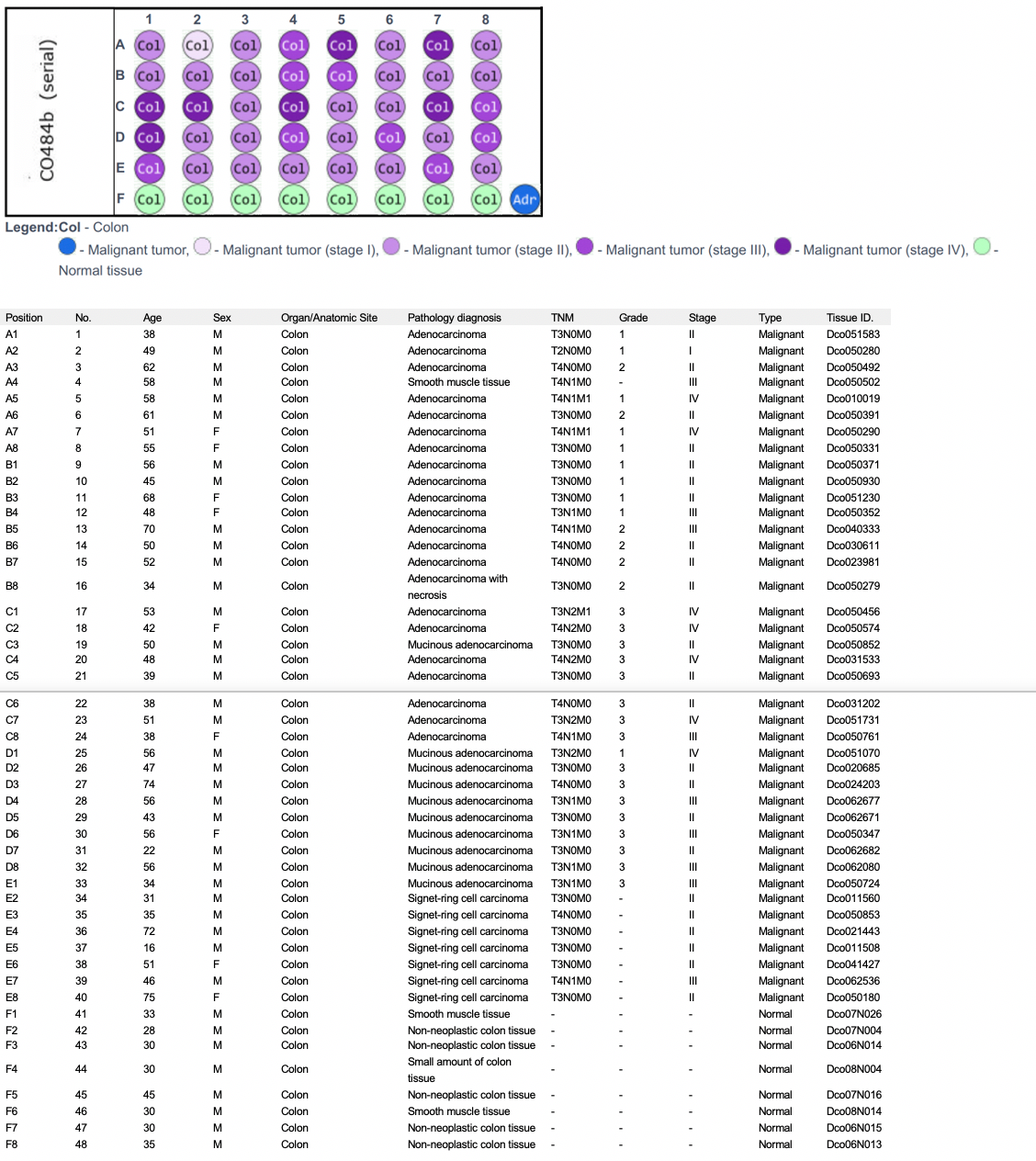
**


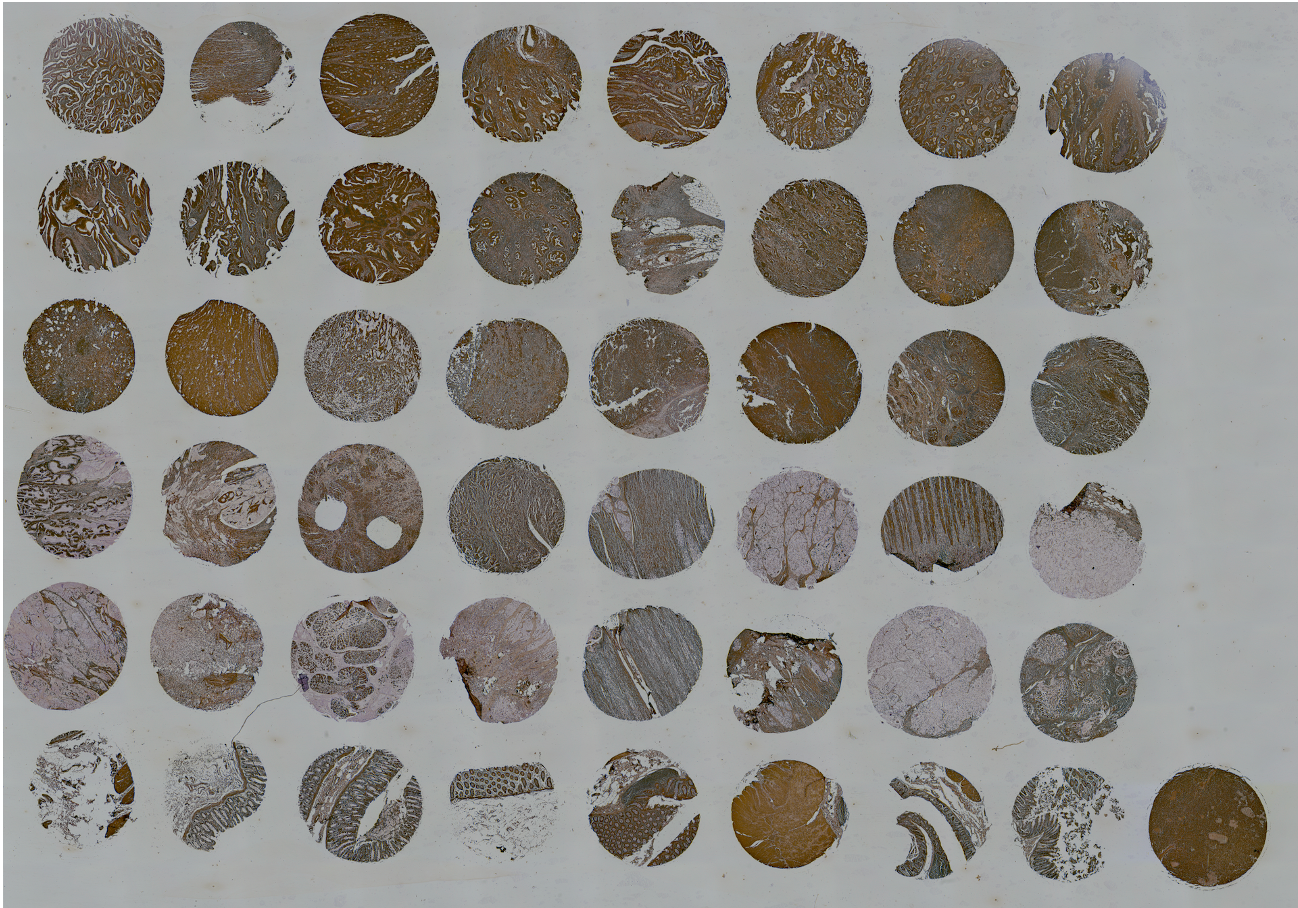


**Figure S6. The expression levels of BRD9 in colon cancer from clinical samples.** The patient details of tissue samples and all imaged taken for immunohistochemistry were included. Sample layouts were shown in the tissue microarray slide.

**
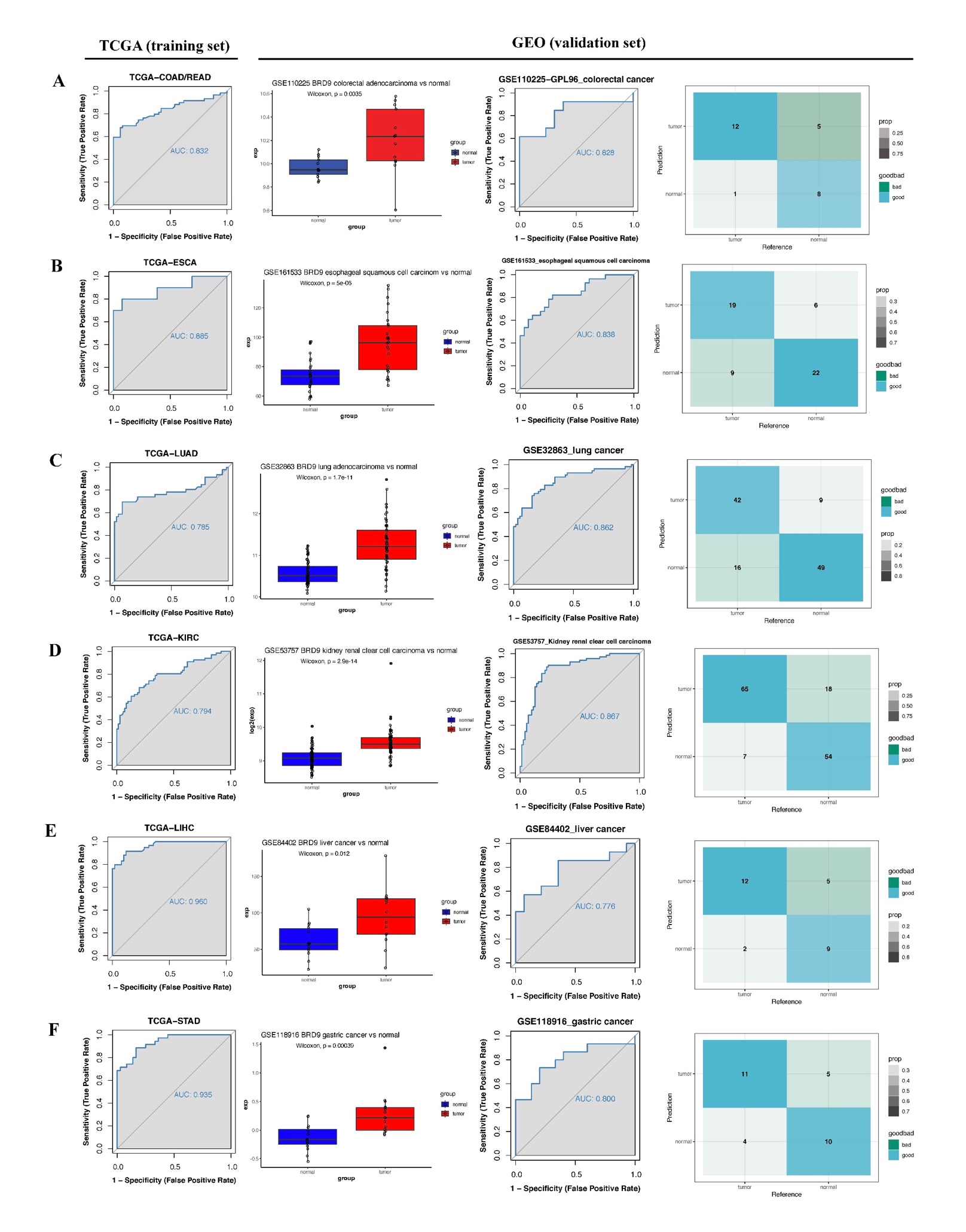
**

**Figure S7. Logistic regression model for independent validation of BRD9 diagnostic ability.**Through receiver operating characteristic (ROC) curve analysis, the potential diagnostic value of *BRD9* was assessed for six cancer types, including colorectal adenocarcinoma (A), esophageal squamous cell carcinoma (B), lung adenocarcinoma (C), kidney renal clear cell carcinoma (D), liver hepatocellular carcinoma (E), and stomach adenocarcinoma (F). Expression profile of *BRD9* in each TCGA cohort was treated as training set, and microarray datasets from GEO corresponding to each cancer type was used as independent validation. Each set of figures included ROC curve based on the TCGA cohort; expression profile of *BRD9*, ROC curve and confusion matrix based on GEO cohort.

**Figure S8. Diagnostic value of *BRD9* in the TCGA cohorts according to ROC curve analysis.** Two cancer types were analyzed, including TCGA-KIRP(A) and TCGA-LUSC (B).

**
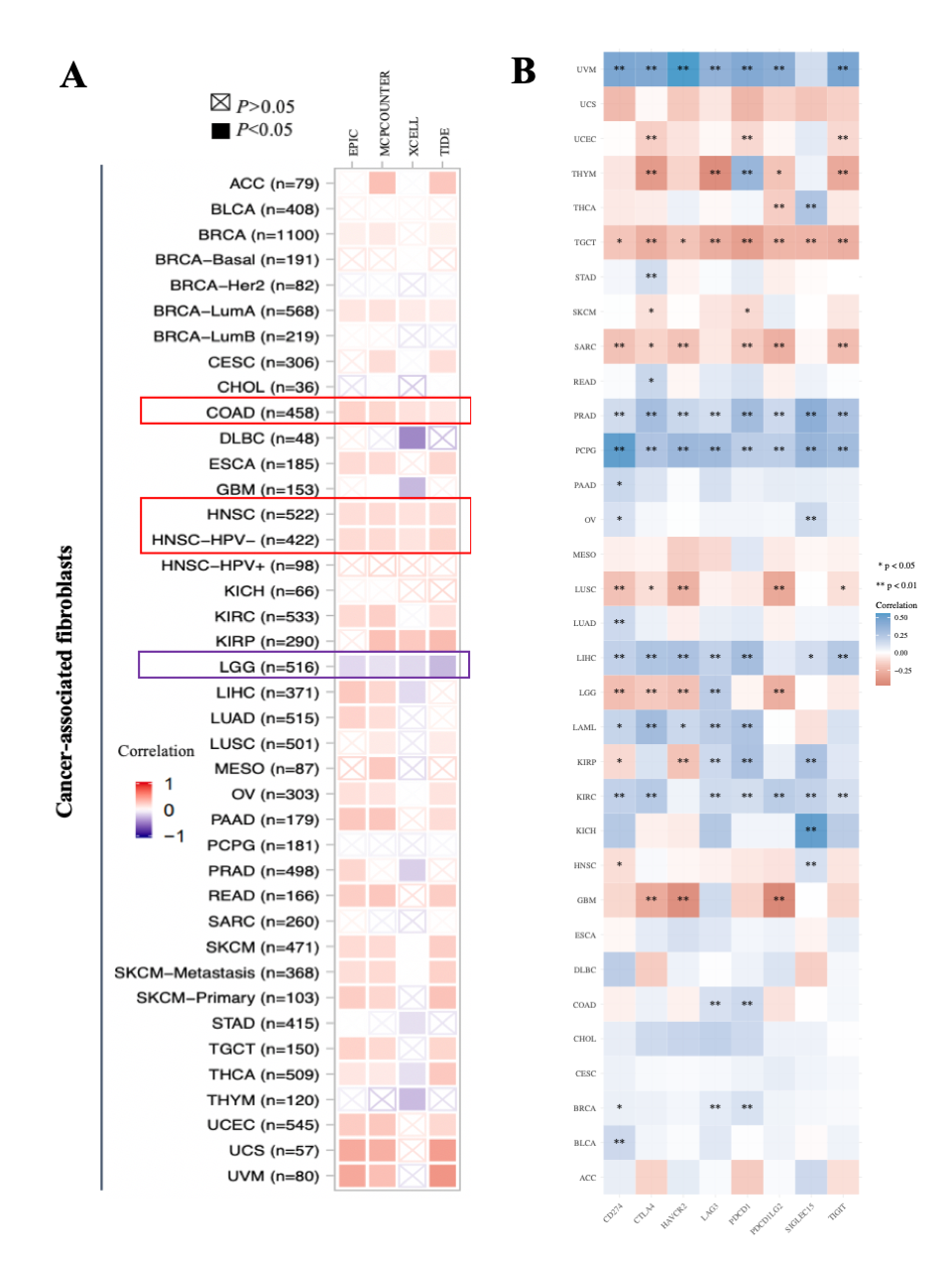
**

**Figure S9.** **Immune-related analysis associated with *BRD9*.** (A) The heatmap showed a correlation analysis between *BRD9* expression and ﻿the estimated abundance of cancer-associated fibroblasts based on EPIC, MCPCOUNTER, XCELL and TIDE algorithms. (B) The correlation analysis between the expression of immune checkpoints and *BRD9* expression in multiple cancers. The immune checkpoints include SIGLEC15, TIGIT, CD274, HAVCR2, PDCD1, CTLA4, LAG3 and PDCD1LG2 (^*^*P* < 0.05, ^**^*P* < 0.01).

**
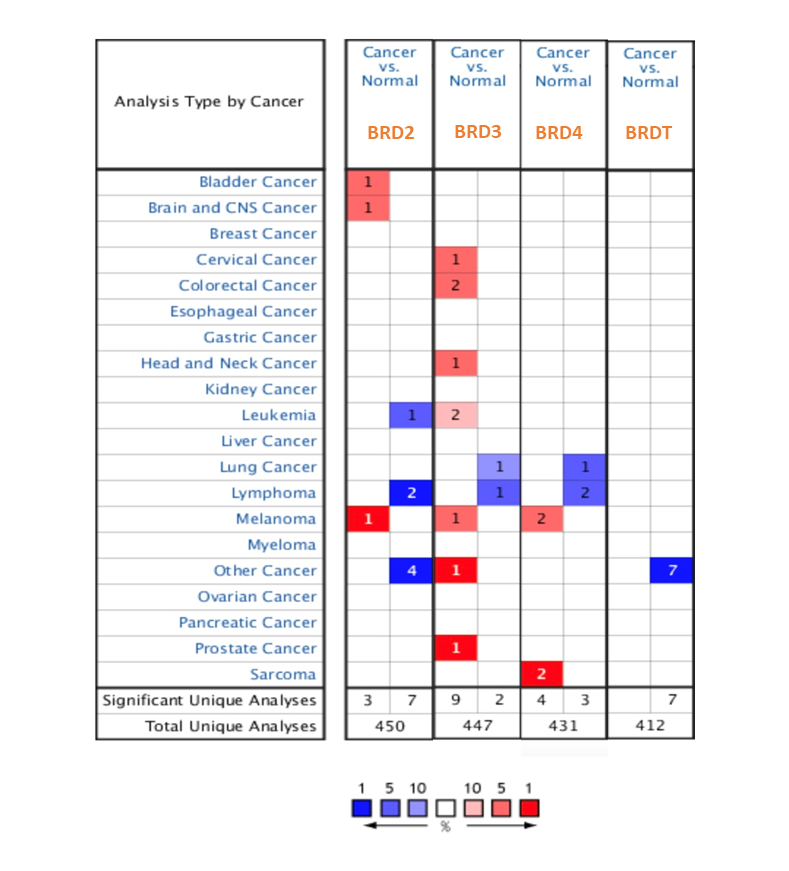
**

**Figure S10.** **The mRNA levels of other BRD family genes in multiple cancers (Oncomine).** It showed the statistically significant other BRD family genes (over-expression (red) or downregulated expression (blue)) by using the numbers of datasets from Oncomine.


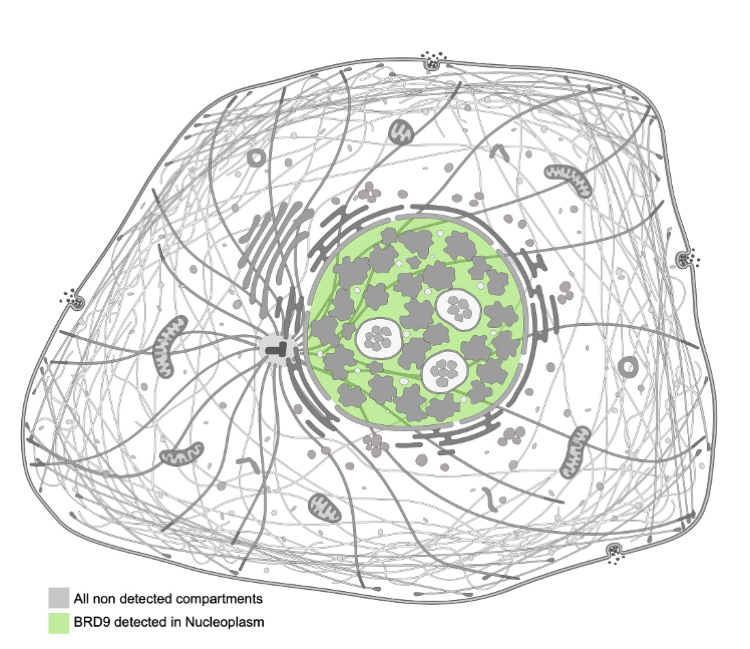


**Figure S11. The subcellular localization of BRD9.** It was detected by the “CELL ATLAS” module of the Human Protein Atlas.

**Figure S12.** **The expression level of each isoform of *BRD*9 in multiple cancers.** The violin-plots presented isoform expression (log2(TPM + 1)).

**Figure S13. The isoform usage of *BRD9* in multiple cancers.** The bar-plot panels revealed isoform usage.

**Figure S14. ﻿Visualization of ﻿the TCGA data for *BRD9* in KIRC by using MEXPRESS.** The highlighted part with a red dashed line in the figure showed *BRD9* DNA methylation level at probes of the promoter region with the Pearson correlation coefficients (*p <0.05, **p <0.01, ***p <0.001).

**Figure S15. ﻿Visualization of ﻿the TCGA data for *BRD9* in KIRP by using MEXPRESS.** The highlighted part with a red dashed line in the figure showed *BRD9* DNA methylation level at probes of the promoter region with the Pearson correlation coefficients (*p <0.05, **p <0.01, ***p <0.001).

**Figure S16. A Landscape of mutation profiles.** In LUSC (A) and UCEC (B) samples based on TCGA database, mutation information of each gene in each sample was shown in the waterfall plot, where different colors with specific annotations at the bottom meant the various mutation types. The bar plot above the legend exhibited the number of mutation burden.

**Figure S17.** **Correlation analysis of *CD274* (*PD-L1*) expression with *BRD9* or *SMARCD1* expression in the TCGA-LIHC (A) and TCGA-MESO (B) cohorts.**

**Supplementary Tables**

**Table S1. *BRD9* expression levels in tumor samples from TCGA cancer types.**

| Tumor type | Abbreviation | Expression level compared with normal samples |
| --- | --- | --- |
| Bladder Urothelial Carcinoma | BLCA | ns |
| Breast invasive carcinoma | BRCA | ns |
| Cervical squamous cell carcinoma and endocervical adenocarcinoma | CESC | ns |
| Cholangiocarcinoma | CHOL | upregulated |
| Colon adenocarcinoma | COAD | upregulated |
| Esophageal carcinoma | ESCA | upregulated |
| Head and Neck squamous cell carcinoma | HNSC | upregulated |
| Kidney Chromophobe | KICH | upregulated |
| Kidney renal clear cell carcinoma | KIRC | upregulated |
| Kidney renal papillary cell carcinoma | KIRP | upregulated |
| Liver hepatocellular carcinoma | LIHC | upregulated |
| Lung adenocarcinoma | LUAD | upregulated |
| Lung squamous cell carcinoma | LUSC | upregulated |
| Pancreatic adenocarcinoma | PAAD | ns |
| Pheochromocytoma and Paraganglioma | PCPG | ns |
| Prostate adenocarcinoma | PRAD | ns |
| Rectum adenocarcinoma | READ | upregulated |
| Sarcoma | SARC | N/A |
| Skin Cutaneous Melanoma | SKCM | N/A |
| Stomach adenocarcinoma | STAD | upregulated |
| Thyroid carcinoma | THCA | ns |
| Thymoma | THYM | N/A |
| Uterine Corpus Endometrial Carcinoma | UCEC | ns |

N/A: not applicable. ns: not significance.

**Table S2. ﻿Genetic alteration of BRD9 in TCGA tumors.**

| Tumor type | Total cases | Total Gene alteration (%) | ﻿Genetic alteration (%) | | | | |
| --- | --- | --- | --- | --- | --- | --- | --- |
|  |  |  | Mutation | Fusion | Amplification | Deep deletion | Multiple alterations |
| Adrenocortical carcinoma | 91 | 13.19% | N/A | N/A | 13.19% (12 cases) | N/A | N/A |
| Bladder Urothelial Carcinoma | 411 | 10.71% | 1.22% (5 cases) | N/A | 9.49% (39 cases) | N/A | N/A |
| Breast invasive carcinoma | 1084 | 2.58% | 0.28% (3 cases) | N/A | 2.21% (24 cases) | 0.09% (1 case) | N/A |
| Cervical squamous cell carcinoma | 297 | 7.07% | 0.34% (1 case) | N/A | 6.4% (19 cases) | 0.34% (1 case) | N/A |
| Colon adenocarcinoma | 594 | 2.36% | 1.52% (9 cases) | N/A | 0.51% (3 cases) | 0.34% (2 cases) | N/A |
| Lymphoid Neoplasm Diffuse Large B-cell Lymphoma | 48 | 2.08% | N/A | N/A | 2.08% (1 case) | N/A | N/A |
| Esophageal adenocarcinoma | 182 | 14.29% | 0.55% (1 case) | N/A | 12.09% (22 cases) | 0.55% (1 case) | 1.1% ( 2 case) |
| Glioblastoma multiforme | 592 | 1.52% | 0.34% (2 cases) | N/A | 0.34% (2 cases) | 0.84% (5 cases) | N/A |
| Head and Neck squamous cell carcinoma | 523 | 5.93% | 0.96% (5 cases) | N/A | 4.97% (26 cases) | N/A | N/A |
| Kidney renal clear cell carcinoma | 511 | 0.98% | N/A | N/A | 0.98% (5 cases) | N/A | N/A |
| Kidney renal papillary cell carcinoma | 283 | 0.71% | N/A | N/A | 0.35% (1 case) | 0.35% (1 case) | N/A |
| Brain Lower Grade Glioma | 514 | 0.97% | N/A | N/A | 0.39% (2 cases) | 0.58% (3 cases) | N/A |
| Liver hepatocellular carcinoma | 372 | 5.11% | 1.08% (4 cases) | N/A | 3.76% (14 cases) | N/A | 0.27% (1 case) |
| Lung adenocarcinoma | 566 | 12.72% | 0.88% (5 cases) | 0.18% (1 case) | 11.66% (66 cases) | N/A | N/A |
| Lung squamous cell carcinoma | 487 | 15.20% | 0.21% (1 case) | 0.21% (1 case) | 14.17% (69 cases) | N/A | 0.62% (3 cases) |
| Mesothelioma | 87 | 1.15% | N/A | N/A | 1.15% (1 case) | N/A | N/A |
| Ovarian serous cystadenocarcinoma | 584 | 9.25% | 0.34% (2 cases) | N/A | 8.9% (52 cases) | N/A | N/A |
| Pancreatic adenocarcinoma | 184 | 0.54% | N/A | N/A | 0.54% (1 case) | N/A | N/A |
| Prostate adenocarcinoma | 494 | 0.61% | 0.2% (1 case) | N/A | 0.4% (2 cases) | N/A | N/A |
| Sarcoma | 255 | 5.49% | N/A | N/A | 4.71% (12 cases) | 0.78% (2 cases) | N/A |
| Skin Cutaneous Melanoma | 444 | 8.33% | 3.83% (17 cases) | 0.23% (1 case) | 3.6% (16 cases) | 0.45% (2 cases) | 0.23% (1 case) |
| Testicular Germ Cell Tumors | 149 | 1.34% | N/A | N/A | N/A | 1.34% (2 cases) | N/A |
| Thyroid carcinoma | 500 | 0.40% | N/A | N/A | 0.4% (2 cases) | N/A | N/A |
| Uterine Corpus Endometrial Carcinoma | 529 | 7.18% | 4.16% (22 cases) | N/A | 2.84% (15 cases) | 0.19% (1 case) | N/A |
| Uterine Carcinosarcoma | 57 | 5.26% | N/A | N/A | 3.51% (2 cases) | 1.75% (1 case) | N/A |

N/A: not applicable.

**Table S3. A series of BRD9-binding proteins supported by experimental evidence were obtained through STRING tool.**

| Gene Symbol | Gene ID |
| --- | --- |
| BCL7A | ENSP00000445868 |
| BCL7C | ENSP00000369674 |
| CATIP | ENSP00000289388 |
| CXXC4 | ENSP00000378248 |
| EPHA1 | ENSP00000275815 |
| EPHA10 | ENSP00000362139 |
| EPHA2 | ENSP00000351209 |
| EPHA3 | ENSP00000337451 |
| EPHA4 | ENSP00000281821 |
| EPHA5 | ENSP00000480763 |
| EPHA6 | ENSP00000374323 |
| EPHA7 | ENSP00000358309 |
| EPHA8 | ENSP00000166244 |
| EPHB1 | ENSP00000381097 |
| EPHB2 | ENSP00000363763 |
| EPHB3 | ENSP00000332118 |
| EPHB4 | ENSP00000350896 |
| EPHB6 | ENSP00000481994 |
| NFE2L1 | ENSP00000354855 |
| PSEN1 | ENSP00000326366 |
| PSEN2 | ENSP00000355747 |
| PTPN13 | ENSP00000394794 |
| SMARCA2 | ENSP00000265773 |
| SMARCA4 | ENSP00000395654 |
| SMARCC2 | ENSP00000267064 |
| SMARCD1 | ENSP00000378414 |
| SPOP | ENSP00000377001 |
| SPOPL | ENSP00000280098 |
| SS18 | ENSP00000414516 |

**Table S4. All tumor expression data of TCGA was integrated and then got the top 100 genes that correlated with *BRD9* expression.**

| Gene Symbol | Gene ID | PCC^a^ |
| --- | --- | --- |
| NSUN2 | ENSG00000037474.14 | 0.65 |
| TRIP13 | ENSG00000071539.13 | 0.62 |
| PAPD7 | ENSG00000112941.12 | 0.61 |
| MED10 | ENSG00000133398.3 | 0.57 |
| ICE1 | ENSG00000164151.11 | 0.57 |
| PDCD6 | ENSG00000249915.7 | 0.56 |
| CCT5 | ENSG00000150753.11 | 0.54 |
| CEP72 | ENSG00000112877.7 | 0.53 |
| BRIX1 | ENSG00000113460.12 | 0.51 |
| ZNF131 | ENSG00000172262.11 | 0.5 |
| NIPBL | ENSG00000164190.16 | 0.5 |
| RAD1 | ENSG00000113456.18 | 0.5 |
| DNAJC21 | ENSG00000168724.14 | 0.49 |
| CLPTM1L | ENSG00000049656.13 | 0.48 |
| DROSHA | ENSG00000113360.16 | 0.48 |
| NUP155 | ENSG00000113569.15 | 0.48 |
| MARCH6 | ENSG00000145495.14 | 0.47 |
| CCDC127 | ENSG00000164366.3 | 0.46 |
| FAM173B | ENSG00000150756.13 | 0.44 |
| DGCR8 | ENSG00000128191.13 | 0.43 |
| WDR70 | ENSG00000082068.8 | 0.43 |
| OTULIN | ENSG00000154124.4 | 0.43 |
| PAIP1 | ENSG00000172239.13 | 0.43 |
| ERCC3 | ENSG00000163161.12 | 0.42 |
| MRPL36 | ENSG00000171421.12 | 0.42 |
| TARS | ENSG00000113407.13 | 0.42 |
| SFSWAP | ENSG00000061936.9 | 0.42 |
| MTA2 | ENSG00000149480.6 | 0.42 |
| SAFB | ENSG00000160633.12 | 0.42 |
| C5orf51 | ENSG00000205765.8 | 0.41 |
| ZC3H18 | ENSG00000158545.15 | 0.41 |
| TARDBP | ENSG00000120948.15 | 0.41 |
| ILF3 | ENSG00000129351.17 | 0.41 |
| SUPT7L | ENSG00000119760.15 | 0.41 |
| SSRP1 | ENSG00000149136.7 | 0.41 |
| SKP2 | ENSG00000145604.15 | 0.41 |
| DDX55 | ENSG00000111364.15 | 0.41 |
| HNRNPA2B1 | ENSG00000122566.20 | 0.4 |
| WRAP73 | ENSG00000116213.15 | 0.4 |
| HCFC1 | ENSG00000172534.13 | 0.4 |
| EWSR1 | ENSG00000182944.17 | 0.4 |
| ATXN7L3 | ENSG00000087152.15 | 0.4 |
| HNRNPM | ENSG00000099783.11 | 0.4 |
| DHX9 | ENSG00000135829.16 | 0.4 |
| SFPQ | ENSG00000116560.10 | 0.4 |
| U2AF2 | ENSG00000063244.12 | 0.4 |
| SNRNP200 | ENSG00000144028.14 | 0.4 |
| C5orf34 | ENSG00000172244.8 | 0.4 |
| SART3 | ENSG00000075856.11 | 0.4 |
| GUSBP1 | ENSG00000183666.16 | 0.39 |
| CHTOP | ENSG00000160679.12 | 0.39 |
| ZDHHC11 | ENSG00000188818.12 | 0.39 |
| NAA40 | ENSG00000110583.12 | 0.39 |
| HNRNPR | ENSG00000125944.18 | 0.39 |
| PGS1 | ENSG00000087157.18 | 0.39 |
| EXOSC10 | ENSG00000171824.13 | 0.39 |
| SRSF4 | ENSG00000116350.15 | 0.38 |
| CSNK1E | ENSG00000213923.10 | 0.38 |
| EMC1 | ENSG00000127463.13 | 0.38 |
| HNRNPH3 | ENSG00000096746.17 | 0.38 |
| NDUFS6 | ENSG00000145494.11 | 0.38 |
| HNRNPDL | ENSG00000152795.17 | 0.38 |
| HNRNPUL2 | ENSG00000214753.2 | 0.38 |
| CDK11B | ENSG00000248333.7 | 0.38 |
| CTD-2083E4.4 | ENSG00000260774.1 | 0.38 |
| PANK4 | ENSG00000157881.13 | 0.38 |
| SMG5 | ENSG00000198952.8 | 0.38 |
| MBD1 | ENSG00000141644.17 | 0.38 |
| DHX16 | ENSG00000204560.9 | 0.37 |
| DHX38 | ENSG00000140829.11 | 0.37 |
| ZNF622 | ENSG00000173545.4 | 0.37 |
| GCN1L1 | ENSG00000089154.10 | 0.37 |
| EP400 | ENSG00000183495.13 | 0.37 |
| ZNF783 | ENSG00000204946.9 | 0.37 |
| SLC7A6OS | ENSG00000103061.11 | 0.37 |
| DUSP12 | ENSG00000081721.11 | 0.37 |
| HNRNPD | ENSG00000138668.18 | 0.37 |
| DDX23 | ENSG00000174243.9 | 0.37 |
| ISY1 | ENSG00000240682.9 | 0.37 |
| NRF1 | ENSG00000106459.14 | 0.37 |
| TRMU | ENSG00000100416.12 | 0.37 |
| DDX39B | ENSG00000198563.13 | 0.37 |
| STRN4 | ENSG00000090372.14 | 0.36 |
| SRSF2 | ENSG00000161547.14 | 0.36 |
| KLHL17 | ENSG00000187961.13 | 0.36 |
| LEMD2 | ENSG00000161904.11 | 0.36 |
| SLC4A1AP | ENSG00000163798.13 | 0.36 |
| KHDRBS1 | ENSG00000121774.17 | 0.36 |
| ANKLE2 | ENSG00000176915.14 | 0.36 |
| E2F4 | ENSG00000205250.8 | 0.36 |
| KDM5C | ENSG00000126012.11 | 0.36 |
| DDX51 | ENSG00000185163.9 | 0.36 |
| UBTF | ENSG00000108312.14 | 0.36 |
| MDC1 | ENSG00000137337.14 | 0.36 |
| RAI14 | ENSG00000039560.13 | 0.36 |
| EXOC3 | ENSG00000180104.15 | 0.36 |
| SMARCD1 | ENSG00000066117.14 | 0.36 |
| FOXK1 | ENSG00000164916.10 | 0.36 |
| STX6 | ENSG00000135823.13 | 0.36 |
| PRKDC | ENSG00000253729.7 | 0.36 |

^a^PCC: Pearson correlation coefficient.

**Table S5. The specific clinical value and the corresponding possible functions/ mechanisms of BRD9 in different cancers.**

| Cancer type | The specific clinical value and the corresponding possible functions/mechanisms of BRD9 | Reference |
| --- | --- | --- |
| Acute leukemia and multiple myeloma | BRD9 degraders may serve as chemosensitizers in acute leukemia and multiple myeloma. | ^1^ |
| Acute leukemia | BRD9 bound cell type-specific chromatin regions regulating leukemic cell survival via STAT5 inhibition. | ^2^ |
| Colorectal cancer | BRD9 was an essential regulator of glycolysis that creates an epigenetic vulnerability in colon adenocarcinoma. | ^3^ |
|  | BRD9 activated proliferation and epithelial-mesenchymal transition of colorectal cancer via the estrogen pathway in vivo and in vitro. | ^4^ |
| Gastric cancer | BRD9 controlled the oxytocin signaling pathway in gastric cancer via CANA2D4, CALML6, GNAO1, and KCNJ5. | ^5^ |
| Gastrointestinal stromal tumor | BRD9 inhibition promoted PUMA-dependent apoptosis and augments the effect of imatinib in gastrointestinal stromal tumors. | ^6^ |
| Hepatocellular carcinoma | BRD9 promoted the growth and metastasis of human hepatocellular carcinoma by activating the TUFT1/AKT pathway. | ^7^ |
|  | BRD9 promoted hepatocellular carcinoma progression via activating the Wnt/β-catenin signaling pathway. | ^8^ |
| Myeloma | BRD9 was essential for ribosome biogenesis and the survival of multiple myeloma cells. | ^9^ |
| Ovarian cancer | BRD9 orchestrated RAD51–RAD54 complex formation and regulated homologous recombination-mediated repair. BRD9 was identified as a potential therapeutic target to promote synthetic lethality and overcome chemoresistance. | ^10^ |
| Prostate cancer | BRD9 was a critical regulator of androgen receptor signaling and prostate cancer progression. | ^11^ |
| Rhabdoid tumor | BRD9 defined a SWI/SNF sub-complex and constituted a specific vulnerability in malignant rhabdoid tumors. | ^12^ |
| Synovial sarcoma | Targeted degradation of BRD9 reversed oncogenic gene expression in synovial sarcoma. | ^13^ |

**Supplementary references**

1. Weisberg E, Chowdhury B, Meng C, et al. BRD9 degraders as chemosensitizers in acute leukemia and multiple myeloma. *Blood Cancer J.* 2022;12(7):110.

2. Del Gaudio N, Di Costanzo A, Liu NQ, et al. BRD9 binds cell type-specific chromatin regions regulating leukemic cell survival via STAT5 inhibition. *Cell Death Dis.* 2019;10(5):338.

3. Zhu Q, Gu X, Wei W, Wu Z, Gong F, Dong X. BRD9 is an essential regulator of glycolysis that creates an epigenetic vulnerability in colon adenocarcinoma. *Cancer Medicine.* 2022;12(2):1572-1587.

4. Chen P, Du R, Chang Z, Gao W, Zhao W, Dong G. Bromodomain-containing protein 9 activates proliferation and epithelial-mesenchymal transition of colorectal cancer via the estrogen pathway in vivo and in vitro. *J Gastrointest Oncol.* 2023;14(2):980-996.

5. Wang Y, Jiang XY, Yu XY. BRD9 controls the oxytocin signaling pathway in gastric cancer via CANA2D4, CALML6, GNAO1, and KCNJ5. *Transl Cancer Res.* 2020;9(5):3354-3366.

6. Mu J, Sun X, Zhao Z, Sun H, Sun P. BRD9 inhibition promotes PUMA-dependent apoptosis and augments the effect of imatinib in gastrointestinal stromal tumors. *Cell Death Dis.* 2021;12(11):962.

7. Dou C, Sun L, Wang L, et al. Bromodomain-containing protein 9 promotes the growth and metastasis of human hepatocellular carcinoma by activating the TUFT1/AKT pathway. *Cell Death Dis.* 2020;11(9):730.

8. Fang D, Wang MR, Guan JL, et al. Bromodomain-containing protein 9 promotes hepatocellular carcinoma progression via activating the Wnt/beta-catenin signaling pathway. *Exp Cell Res.* 2021;406(2):112727.

9. Kurata K, Samur MK, Liow P, et al. BRD9 Is Essential for Ribosome Biogenesis and the Survival of Multiple Myeloma Cells. *Blood.* 2022;140(Supplement 1):596-597.

10. Zhou Q, Huang J, Zhang C, et al. The bromodomain containing protein BRD-9 orchestrates RAD51-RAD54 complex formation and regulates homologous recombination-mediated repair. *Nat Commun.* 2020;11(1):2639.

11. Alpsoy A, Utturkar SM, Carter BC, et al. BRD9 Is a Critical Regulator of Androgen Receptor Signaling and Prostate Cancer Progression. *Cancer Res.* 2021;81(4):820-833.

12. Wang X, Wang S, Troisi EC, et al. BRD9 defines a SWI/SNF sub-complex and constitutes a specific vulnerability in malignant rhabdoid tumors. *Nat Commun.* 2019;10(1):1881.

13. Brien GL, Remillard D, Shi J, et al. Targeted degradation of BRD9 reverses oncogenic gene expression in synovial sarcoma. *Elife.* 2018;7.
