# Supplementary material for: Pan‐cancer analyses of bromodomain containing 9 as a novel therapeutic target reveals its diagnostic, prognostic potential and biological mechanism in human tumours
Source: Clin Transl Med. 2024 Feb 1;14(2):e1543. doi: 10.1002/ctm2.1543 (PMC10835192; doi:10.1002/ctm2.1543)
Supplement: Supplementary file 2 — Supporting information [file CTM2-14-e1543-s002.docx]

**Supplementary Materials and Methods**

**1. Experimental validation**

**1.1 Cell culture**

Human lung adenocarcinoma cell line A549 and human normal lung cell line MRC-9 were purchased from European Collection of Authenticated Cell Cultures (ECACC). Human lung adenocarcinoma cell line ABC-1 and human lung squamous cell carcinoma cell lines EBC-1 and LK-2 were purchased from Japanese Collection of Research Bioresources (JCRB) Cell Bank. Human normal lung cell lines HLF and BEAS-2B, and human normal colon cell line were purchased from American Type Culture Collection (ATCC). Human colon cancer cell lines HT-29, RKO, SW480, CaCo2, and HCT116 were gifts from Dr. Peiwen Fei’s lab. Lung Fibroblast HLF maintained in fibroblast basal medium was supplemented with fibroblast growth kit–Low Serum (ATCC, USA), and 5,000 U/mL Penicillin-Streptomycin (Gibco, ThermoFisher, USA) in humidified air at 37 °C with 5% CO2. The other cells were maintained in Dulbecco’s modified Eagle medium (DMEM, Gibco, USA) supplemented with 10% fetal bovine serum (FBS) (Sigma-Aldrich, St. Louis, MO, USA), and 5,000 U/mL Penicillin-Streptomycin (Gibco, ThermoFisher, USA) in humidified air at 37 °C with 5% CO2.

**1.2 Cell transfection**

﻿The cells were transiently transfected for 48h with 20 nM siRNA against *BRD9* (human Hs_*BRD9*_6 FlexiTube siRNA, SI03228498; human Hs_*BRD9*_12 FlexiTube siRNA, SI05169465), or scrambled sequences (Negative Control siRNA, 1027310) using HiPerFect Transfection Reagent (QIAGEN, USA). *BRD9* siRNA and mismatch siRNA were synthesized by QIAGEN and annealed to form duplexes. Two *BRD9*-specifc siRNA sequences were used: siRNA#1, 5′-TAGGCCAGATACCGTGTACTA-3′; siRNA#2, 5′- CACGCTGGGCTTCAAAGACGA-3′.

**1.3 RNA extraction and quantitative PCR analysis**

Total RNA was isolated using Direct-zol RNA Miniprep Plus Kits (Zymo research, USA). Complementary DNA (cDNA) was synthesized using PrimeScript™ RT reagent Kit (Perfect Real Time) (TaKaRa, Japan). Quantitative PCR was performed using TaqMan gene expression assay (Hs01079464_g1, ThermoFisher, USA). GAPDH was used as an internal control for mRNA. Relative RNA abundances were calculated using the standard 2-ΔΔCt method.

**1.4 Cell proliferation assay**

The cell proliferation assay was performed using the Cell Counting Kit-8 (WST-8, ab228554; Abcam, USA) according to the manufacturer’s instructions. Lung cancer and colon cancer cells were seeded into 96-well plates (5000 cells per well). After being cultivated in the incubator for 24, 48 and 72 h, cells were incubated with 10 μl WST-8 solution for 1 h. Afterwards, the absorbance was measured at 460 nm.

**1.5 Apoptosis analyzed by flow cytometry**

Cell apoptosis was measured using the FITC Annexin V Apoptosis Detection Kit I (RUO) (556547, BD Pharmingen TM, USA). Cells were harvested and washed twice with cold PBS and resuspended in 100 μl 1× binding buffer. 5 μl FITC Annexin V and 5 μl propidium iodide (PI) were added to each group and incubated in the dark for 15 min at room temperature. 30,000 cells of each group were measured by a Accuri C6 Flow Cytometer (Becton Dickenson, USA) and then analyzed data with the FlowJo 7.6 software.

**1.6 Colony formation assay**

500 cells were seeded per well in six-well plates. After 2 weeks, cell colonies were washed with PBS, fixed with 4% formaldehyde solution for 15 min and stained with crystal violet for 20 min. The colonies were imaged and counted.

**1.7 Immunofluorescence**

The cells were cultured in complete cell culture medium on glass coverslips in 24-well plates for 24h, washed with PBS three times, then fixed in 4% formaldehyde solution for 15 min. The cells were permeabilized using 0.5% Triton X-100 in PBS at room temperature for 10 min and blocked with 5% BSA solution at 37 °C for 1 h. The cells were incubated overnight with anti-BRD9 antibody (PA5-113488, Thermo Fisher) and anti-SMARCD1 antibody (sc-135843, Santa cruz) at 4 °C. After being washed, the bound antibodies were reacted with a corresponding Alexa Fluor® conjugated secondary antibody (8889S, 4408S, CST) at room temperature for 1 h and nuclear-counterstained with 4′,6-diamidino-2-phenylindole (DAPI, 62248, Thermo Fisher, USA). The immunofluorescent images were obtained by using a Leica SP5 confocal microscope.

**1.8 Western blot**

﻿The proteins were extracted from cells and then separated by mPAGE® 4-12% Bis-Tris Precast Gel before being transferred to 0.45 μm PVDF membranes (Millipore, USA). The PVDF membranes were blocked with 5% BSA solution and incubated with specific primary antibodies at 4 °C overnight. The PVDF membranes were then incubated with the appropriate secondary antibodies, and Odyssey® DLx Imaging System (LI-COR, USA) was used to detect the protein bands. β-Actin was used as a control.

**1.9 Immunochemistry**

Non-small cell lung cancer (NSCLC) tissue arrays with normal lung tissue (LC10011b) were purchased from AMSBIO. The tissue microarray contained 24 cases of squamous cell carcinoma, 16 adenocarcinoma, 10 normal lung tissue, duplicate cores per case, divided into two identical 50-core arrays. Colon cancer tissue arrays with adjacent normal tissues (CO484b) were also purchased from AMSBIO. The tissue microarrays contained 40 cases of colon adenocarcinoma, plus 8 normal colon tissue, single core per case. The array slides were deparaffinized and dried. Then, array slides were rinsed twice with PBS for 5 min each. The endogenous peroxidase activity was blocked at room temperature by a 5-10 min incubation in the final developmental 3% H2O2 in PBS (pH 7.4). The array slides were rinsed in PBS for 5 min. Antigen retrieval was performed. The array slides were rinsed again in PBS for 5 min. The blocking antibody (normal goat serum) was applied, incubated for 20 min at room temperature, and residual fluid was thrown off. The BRD9 primary antibody was applied at 4°C overnight. The array slides were rinsed twice for 5 min each. The array slides were incubated with a biotin-conjugated secondary antibody at 20-37°C for 20 min. The array slides were rinsed twice for 5min each. The array slides were then incubated with SABC reagent at 37°C for 20 min before being rinsed 4 times for 5 min each. The DAB kit was used, and the array slides were washed in distilled water. The array slides were then stained in hematoxylin and mounted.

**1.10 Co-immunoprecipitation (Co-IP)**

﻿Pierce™ Classic Magnetic IP/Co-IP Kit (Thermo Scientific, USA) was used for co-immunoprecipitation assay. Briefly, the specific primary antibody was first added to the cell lysate to form an immune complex that was then bound to the magnetic beads. The complex was washed to remove non-bound material and a low pH elution buffer dissociated the bound immune complex from the Protein A/G. The immune complex was then applied to western blot.

**2. Tumor mutation burden (TMB)/microsatellite instability (MSI) analysis**

To identify the correlation between *BRD9* expression and TMB/MSI in all tumors of TCGA, the results were visualized using the “TCGAmutations” package in R software (v 4.0.3) ^1^.

**3. Total protein/phosphoprotein analysis of BRD9**

Compared with normal tissues, the total protein expression levels or phosphorylation levels (with phosphorylation at the T103, S482, S568, S588 sites) of BRD9 (NP_076413.3) in primary tumor tissues was analyzed using the UALCAN portal (<http://ualcan.path.uab.edu/analysis-prot.html>) ^2^. Six cancer types were available in this ﻿proteomic database, including breast cancer, clear cell renal cell carcinoma (RCC), lung adenocarcinoma (LUAD), ovarian cancer, uterine corpus endometrial carcinoma (UCEC) and colon cancer.

**4. DNA methylation analysis**

The methylation level of *BRD9*, across various tumor types from the TCGA database was analyzed using the β-value of the CpG-aggregated methylation value. Compared with normal tissues, the *BRD9* DNA methylation levels in primary tumor tissues were plotted as box plots using the Shiny Methylation Analysis Resource Tool (SMART) (<http://www.bioinfo-zs.com/smartapp/>) with the settings (e.g., CpG aggregation: all, aggregation method: mean, methylation value: β-value) ^3^. ﻿The *p*-value was calculated using Wilcoxon rank sum test and adjusted using the Benjamini-Hochberg method. DNA methylation data based on TCGA database was visualized as heatmaps for *BRD9* by the “Gene visualization” module of MethSurv (<https://biit.cs.ut.ee/methsurv/>) ^4^. The DNA methylation pattern was annotated by different probes. The DNA methylation level of each probe was calculated by the β‐value (0-1). β-value is one of the indicators used to measure the level of DNA methylation ^5^, with values greater than 0.6 considered to be highly methylated and values less than 0.2 are considered unmethylated. β-values falling between 0.2 and 0.6 are typically considered partially methylated ^6^.

**5. *BRD9*-related gene set enrichment analysis**

BRD9-binding proteins were obtained using the STRING tool ﻿(<https://string-db.org/>), which is a bioinformatics tool to predict protein–protein interactions supported by experimental evidence. The top 100 BRD9-correlated targeting genes among all TCGA tumor and normal tissues was then obtained by using the “Similar Gene Detection” panel of GEPIA 2.

A pairwise gene Pearson correlation analysis of *BRD9* and selected genes was performed by using the “correlation analysis” panel of GEPIA2. The *p*-value and correlation coefficient (R) were shown in the dot plot (log2TPM). In addition, the corresponding heatmap was obtained from the “Exploration-Gene_Corr” panel of TIMER 2.0. Intersection of BRD9-binding and interacting genes were then analyzed using a Venn diagram (Jvenn) ^7^.

These two gene lists were combined and uploaded to the Database for Annotation, Visualization and Integrated Discovery (DAVID) v6.8 ^8^ with the settings of selected identifier (“OFFICIAL_GENE_SYMBOL”) and species (“*Homo sapiens*”). The resultant gene list was then analyzed with DAVID’s functional annotation tool. For Gene Ontology (GO) and Kyoto Encyclopedia of Gene and Genomes (KEGG) pathway enrichment analysis, the data of “GOTERM_BP_DIRECT”, “GOTERM_CC_ DIRECT”, “GOTERM_MF_DIRECT” and “KEGG_PATHWAY” were visualized with the “ggplot2” and “clusterProfiler” R package ^9^.

**6. Expression validation of BRD9**

Six types of cancer shown significantly differential expression from TCGA pan cancer analysis including colorectal adenocarcinoma, esophageal squamous cell carcinoma, lung adenocarcinoma, kidney renal clear cell carcinoma, liver hepatocellular carcinoma, and stomach adenocarcinoma were selected for further validation. Six independent microarray datasets from Gene Expression Omnibus (GEO) were collected. Microarray data were processed by the R package “GEOquery”, and low expression genes were removed. Significant genes were calculated by linear model by R package “limma” and visualization were done by R package “ggplot2”.

**7. Logistic regression model for independent validation of *BRD9* diagnostic ability**

Six types of cancer were used for validation through logistic regression model. Batch effects were removed by “limma” package. Expression profile of *BRD9* in each TCGA cohort was treated as training set, and microarray dataset from GEO corresponding to each cancer type was used as independent validation. The optimal threshold was determined by Youden’s index and receiver operating characteristic (ROC) curve were generated by “pROC” package ^10^.

In addition to the logistic regression validation for *BRD9*, we also used confusion matrix to evaluate the performance of this classification model to understand how well the model could distinguish normal and cancer in different types of cancer based on optimal threshold ^11^. The confusion matrix is composed of four components: true positives (TP), located in the upper left quadrant, which count the number of accurately predicted positive cases; true negatives (TN), found in the lower right quadrant, indicating the correctly predicted negative cases; false positives (FP), in the lower left, which are positive cases incorrectly predicted; and false negatives (FN), in the upper right, representing negative cases that were mistakenly predicted as positive.

**8. Immune correlation analysis associated with *BRD9***

The heatmap were shown to investigate the potential relationship between the prominence of cancer-associated fibroblasts (CAFs) and *BRD9* gene expression in diverse cancer types of TCGA by the “Immune-Gene” module of the TIMER2.0 ^12^. EPIC, MCPCOUNTER, XCELL and TIDE algorithms were used to estimate immune infiltration.

RNA-seq data (TCGA) (<https://portal.gdc.cancer.gov/>) were downloaded from the Genomic Data Commons (GDC) data portal website for 33 cancer types. For reliable immune score evaluation, we used immunedeconv ^13^, an R software package that integrates six latest algorithms, including TIMER, xCell, MCP-counter, CIBERSORT, EPIC and quanTIseq. SIGLEC15, TIGIT, CD274, HAVCR2, PDCD1, CTLA4, LAG3 and PDCD1LG2 are transcripts related to immune checkpoints ^14^. Observations were based on extracted expression values of these 8 genes. R ﻿language software v4.0.3 was used for statistical analysis. The rank sum test detected two sets of data, and a *P* value of <0.05 was considered statistically significant.

**9. Oncomine Analysis**

Oncomine datasets (https://www.oncomine.org/resource/login.html) ^15^ were used to analyze the transcription levels of BET family proteins in pan-cancer. Compared with normal controls, the mRNA expressions of BET family proteins were detected in cancer samples by using a Student’s *t* test (p <0.01, fold change=2).

**10.Subcellular localization**

The subcellular localization of BRD9 was detected by the “CELL ATLAS” module of the Human Protein Atlas ([www.proteinatlas.org](http://www.proteinatlas.org)) ^16^.

**11. Isoform analysis**

The data of ﻿isoform analysis for *BRD9* were obtained by using the “Isoform Details” panel of GEPIA 2 ^17^. The expression level of each isoform of *BRD*9 in pan-cancer was presented as violin-plots (log2(TPM + 1)). The bar-plot panel revealed the isoform usage of *BRD9* in pan-cancer analysis.

**12. Exploring DNA methylation level in the promoter region of *BRD9***

The DNA methylation level in the promoter region of *BRD9* was analyzed by MEXPRESS web server (<https://mexpress.be/>) ^18,19^. Pearson correlation was calculated to compare two types of data that both have more than 2 levels (e.g. expression and methylation data). The Wilcoxon’s rank-sum test ﻿was used to calculate the difference of a variable between two groups (e.g. the difference in expression between male and female). A false discovery rate correction step was used to correct for multiple comparisons.

**13. The landscape of BRD9 mutations analysis**

To identify the somatic mutations of the patients with LUSC and UCEC in the TCGA database, genetic mutation data, transcriptome data, and clinical data were downloaded and visualized using the “maftools” package in R ﻿language software v4.0.3 ^20^.

**14. Statistical analysis**

Statistical analysis was performed using GraphPad Prism 8.0. All comparisons between samples were presented as mean ± standard error of the mean (SEM) or standard deviation (SD), as appropriate. The statistical analyses were conducted using one-way or two-way ANOVA with Dunnett’s, Tukey’s or Sidak’s multiple comparison test, as relevant. Where applicable, statistical significance is denoted by * for P<0.05; **: P<0.01; ***: P<0.001.

**Supplementary figure legends**

**Figure S1. The correlation between BRD9 expression and TMB (A)/MSI (B) across all tumors of TCGA.**

**Figure S2. Protein phosphorylation analysis of BRD9 in different tumors by using CPTAC dataset from UALCAN tool.** The schematic diagram (A) summarized the phosphoprotein sites with positive results for BRD9 (all p <0.05). BRD9 phosphoprotein expression (T103, S482, S568 and S588 sites) between normal tissue and primary tissue of breast cancer (B), ovarian cancer (C), clear cell RCC (D), colon cancer (E), UCEC (F) were analyzed (all p <0.05). Data on LUAD was not shown because there was no significant difference (p >0.05).

**Figure S3. BRD9 DNA methylation analysis in different patient cohorts from TCGA.** (A) The methylation level of BRD9 was analyzed in different tumors from the TCGA database. (B-I) The 8 cancer types with the most significant differences in (A) were selected to draw heatmaps for multiple probes (all P<0.0001). ns: not significant. * p < 0.05; ** P < 0.01; *** p < 0.001; **** p < 0.0001.

**Figure S4. BRD9-related gene set enrichment analysis.** (A) A series of BRD9-binding proteins supported by experimental evidence were obtained using the STRING tool. (B) The top 100 BRD9-correlated genes were obtained from the TCGA database. The correlation of gene expression between *BRD9* and the top 4 targeting genes, including MED10, NSUN2, PAPD7 and TRIP13, were analyzed. (C) The heatmap showed the correlation. Based on *BRD9*-binding genes and highly correlated genes, intersection analysis (D), KEGG pathway analysis (E) and GO analysis (F) were conducted.

**Figure S5. The expression levels of BRD9 in lung cancer from clinical samples.** The patient details of tissue samples and all imaged taken for immunohistochemistry were included. Sample layouts were shown in the tissue microarray slide.

**Figure S6. The expression levels of BRD9 in colon cancer from clinical samples.** The patient details of tissue samples and all imaged taken for immunohistochemistry were included. Sample layouts were shown in the tissue microarray slide.

**Figure S7. Logistic regression model for independent validation of BRD9 diagnostic ability.**Through receiver operating characteristic (ROC) curve analysis, the potential diagnostic value of *BRD9* was assessed for six cancer types, including colorectal adenocarcinoma (A), esophageal squamous cell carcinoma (B), lung adenocarcinoma (C), kidney renal clear cell carcinoma (D), liver hepatocellular carcinoma (E), and stomach adenocarcinoma (F). Expression profile of *BRD9* in each TCGA cohort was treated as training set, and microarray datasets from GEO corresponding to each cancer type was used as independent validation. Each set of figures included ROC curve based on the TCGA cohort; expression profile of *BRD9*, ROC curve and confusion matrix based on GEO cohort.

**Figure S8. Diagnostic value of *BRD9* in the TCGA cohorts according to ROC curve analysis.** Two cancer types were analyzed, including TCGA-KIRP(A) and TCGA-LUSC (B).

**Figure S9.** **Immune-related analysis associated with *BRD9*.** (A) The heatmap showed a correlation analysis between *BRD9* expression and ﻿the estimated abundance of cancer-associated fibroblasts based on EPIC, MCPCOUNTER, XCELL and TIDE algorithms. (B) The correlation analysis between the expression of immune checkpoints and *BRD9* expression in multiple cancers. The immune checkpoints include SIGLEC15, TIGIT, CD274, HAVCR2, PDCD1, CTLA4, LAG3 and PDCD1LG2 (^*^*P* < 0.05, ^**^*P* < 0.01).

**Figure S10.** **The mRNA levels of other BRD family genes in multiple cancers (Oncomine).** It showed the statistically significant other BRD family genes (over-expression (red) or downregulated expression (blue)) by using the numbers of datasets from Oncomine.

**Figure S11. The subcellular localization of BRD9.** It was detected by the “CELL ATLAS” module of the Human Protein Atlas.

**Figure S12.** **The expression level of each isoform of *BRD*9 in multiple cancers.** The violin-plots presented isoform expression (log2(TPM + 1)).

**Figure S13. The isoform usage of *BRD9* in multiple cancers.** The bar-plot panels revealed isoform usage.

**Figure S14. ﻿Visualization of ﻿the TCGA data for *BRD9* in KIRC by using MEXPRESS.** The highlighted part with a red dashed line in the figure showed *BRD9* DNA methylation level at probes of the promoter region with the Pearson correlation coefficients (*p <0.05, **p <0.01, ***p <0.001).

**Figure S15. ﻿Visualization of ﻿the TCGA data for *BRD9* in KIRP by using MEXPRESS.** The highlighted part with a red dashed line in the figure showed *BRD9* DNA methylation level at probes of the promoter region with the Pearson correlation coefficients (*p <0.05, **p <0.01, ***p <0.001).

**Figure S16. A Landscape of mutation profiles.** In LUSC (A) and UCEC (B) samples based on TCGA database, mutation information of each gene in each sample was shown in the waterfall plot, where different colors with specific annotations at the bottom meant the various mutation types. The bar plot above the legend exhibited the number of mutation burden.

**Figure S17.** **Correlation analysis of *CD274* (*PD-L1*) expression with *BRD9* or *SMARCD1* expression in the TCGA-LIHC (A) and TCGA-MESO (B) cohorts.**

**Supplementary Table legends**

**Table S1. *BRD9* expression levels in tumor samples from TCGA cancer types.**

**Table S2. ﻿Genetic alteration of BRD9 in TCGA tumors.**

**Table S3. A series of BRD9-binding proteins supported by experimental evidence were obtained through STRING tool.**

**Table S4. All tumor expression data of TCGA was integrated and then got the top 100 genes that correlated with *BRD9* expression. PCC: Pearson correlation coefficient.**

**Table S5. The specific clinical value and the corresponding possible functions/ mechanisms of BRD9 in different cancers.**

**Supplementary references**

1. Ellrott K, Bailey MH, Saksena G, et al. Scalable Open Science Approach for Mutation Calling of Tumor Exomes Using Multiple Genomic Pipelines. *Cell Syst.* 2018;6(3):271-281 e277.

2. Chen F, Chandrashekar DS, Varambally S, Creighton CJ. Pan-cancer molecular subtypes revealed by mass-spectrometry-based proteomic characterization of more than 500 human cancers. *Nat Commun.* 2019;10(1):5679.

3. Li Y, Ge D, Lu C. The SMART App: an interactive web application for comprehensive DNA methylation analysis and visualization. *Epigenetics Chromatin.* 2019;12(1):71.

4. Modhukur V, Iljasenko T, Metsalu T, Lokk K, Laisk-Podar T, Vilo J. MethSurv: a web tool to perform multivariable survival analysis using DNA methylation data. *Epigenomics.* 2018;10(3):277-288.

5. Xie C, Leung YK, Chen A, Long DX, Hoyo C, Ho SM. Differential methylation values in differential methylation analysis. *Bioinformatics.* 2019;35(7):1094-1097.

6. Novakovic B, Yuen RK, Gordon L, et al. Evidence for widespread changes in promoter methylation profile in human placenta in response to increasing gestational age and environmental/stochastic factors. *BMC Genomics.* 2011;12:529.

7. Bardou P, Mariette J, Escudie F, Djemiel C, Klopp C. jvenn: an interactive Venn diagram viewer. *BMC Bioinformatics.* 2014;15:293.

8. Huang da W, Sherman BT, Lempicki RA. Bioinformatics enrichment tools: paths toward the comprehensive functional analysis of large gene lists. *Nucleic Acids Res.* 2009;37(1):1-13.

9. Yu G, Wang LG, Han Y, He QY. clusterProfiler: an R package for comparing biological themes among gene clusters. *OMICS.* 2012;16(5):284-287.

10. Robin X, Turck N, Hainard A, et al. pROC: an open-source package for R and S+ to analyze and compare ROC curves. *BMC Bioinformatics.* 2011;12:77.

11. Stehman SV. Selecting and interpreting measures of thematic classification accuracy. *Remote Sensing of Environment.* 1997;62(1):77-89.

12. Li T, Fu J, Zeng Z, et al. TIMER2.0 for analysis of tumor-infiltrating immune cells. *Nucleic Acids Res.* 2020;48(W1):W509-W514.

13. Sturm G, Finotello F, Petitprez F, et al. Comprehensive evaluation of transcriptome-based cell-type quantification methods for immuno-oncology. *Bioinformatics.* 2019;35(14):i436-i445.

14. Deng C, Guo H, Yan D, et al. Pancancer Analysis of Neurovascular-Related NRP Family Genes as Potential Prognostic Biomarkers of Bladder Urothelial Carcinoma. *BioMed Research International.* 2021;2021:1-31.

15. Rhodes DR, Kalyana-Sundaram S, Mahavisno V, et al. Oncomine 3.0: genes, pathways, and networks in a collection of 18,000 cancer gene expression profiles. *Neoplasia.* 2007;9(2):166-180.

16. Ponten F, Jirstrom K, Uhlen M. The Human Protein Atlas--a tool for pathology. *J Pathol.* 2008;216(4):387-393.

17. Tang Z, Kang B, Li C, Chen T, Zhang Z. GEPIA2: an enhanced web server for large-scale expression profiling and interactive analysis. *Nucleic Acids Res.* 2019;47(W1):W556-W560.

18. Koch A, De Meyer T, Jeschke J, Van Criekinge W. MEXPRESS: visualizing expression, DNA methylation and clinical TCGA data. *BMC Genomics.* 2015;16:636.

19. Koch A, Jeschke J, Van Criekinge W, van Engeland M, De Meyer T. MEXPRESS update 2019. *Nucleic Acids Res.* 2019;47(W1):W561-W565.

20. Mayakonda A, Lin D-C, Assenov Y, Plass C, Koeffler HP. Maftools: efficient and comprehensive analysis of somatic variants in cancer. *Genome Research.* 2018;28(11):1747-1756.
